# Supplementary material for: Opposite physiological and pathological mTORC1-mediated roles of the CB1 receptor in regulating renal tubular function
Source: Nat Commun. 2022 Apr 4;13:1783. doi: 10.1038/s41467-022-29124-8 (PMC8980033; doi:10.1038/s41467-022-29124-8)
Supplement: Supplementary file 1 — Supplementary Information [file 41467_2022_29124_MOESM1_ESM.pdf]

SUPPLEMENTARY INFORMATION

Opposite Physiological and Pathological mTORC1-mediated Roles of the CB1 Receptor in Regulating Renal Tubular Function

Liad Hinden[1], Majdoleen Ahmad[1], Sharleen Hamad[1], Alina Nemirovski[1], Gergő Szanda[2], Sandra Glasmacher[3], Aviram Kogot-Levin[4], Rinat Abramovitch[5,6], Bernard Thorens[7], Jürg Gertsch[3], Gil Leibowitz[4], and Joseph Tam[1*]

[*]Correspondence to: yossi.tam@mail.huji.ac.il

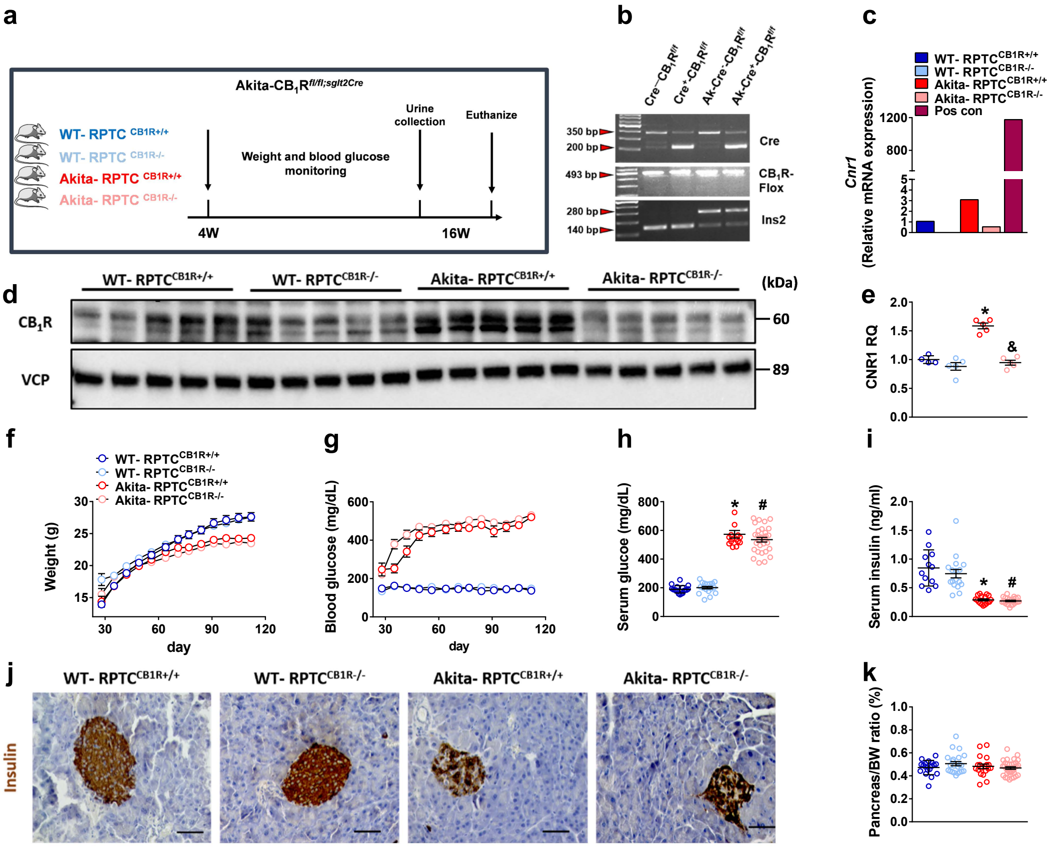

Supplementary Fig 1. Characterization of the Akita-RPTC$^{CB1R-/-}$ diabetic mouse model, related to Fig 1, 2, and 4.

(a) Scheme of the experimental paradigm using the WT and Akita mice, with or without the presence of CB_1R. The illustration of the mice was prepared using Servier Medical Art website (smart.servier.com).

(b) Typical genotyping of each experimental group for Cre recombinase, CB_1R-flox, and Ins2.

(c) qPCR analysis of the kidney CB_1R (*Cnr1*) mRNA expression levels in each of the experimental groups compared to the brain mRNA levels as a positive control (Pos Con). n=2 replicates. The mRNA expression level of target genes was normalized to that of *Ubc* (ubiquitin C).

(d, e) Immunoblotting analysis and quantification of kidney CB_1R (CNR1) protein in cortical kidney lysates from each experimental group. n=5 mice per group (*P<0.0001, $^{\&}$P<0.0001).

(f) Body weight surveillance for 16 weeks. n=20 mice for WT-RPTC$^{CB1R+/+}$, n=23 mice for WT-RPTC$^{CB1R-/-}$, n=20 mice for Akita-RPTC$^{CB1R+/+}$, and n=34 mice for Akita-RPTC$^{CB1R-/-}$.

(g) Blood glucose surveillance for 16 weeks. n=20 mice for WT-RPTC$^{CB1R+/+}$, n=23 mice for WT-RPTC$^{CB1R-/-}$, n=20 mice for Akita-RPTC$^{CB1R+/+}$, and n=34 mice for Akita-RPTC$^{CB1R-/-}$.

(h) Serum glucose levels at 16 weeks of age. n=18 mice for WT-RPTC$^{CB1R+/+}$, n=19 mice for WT-RPTC$^{CB1R-/-}$, n=15 mice for Akita-RPTC$^{CB1R+/+}$, and n=30 mice for Akita-RPTC$^{CB1R-/-}$ (*P<0.0001, $^{\#}$P<0.0001).

(i) Serum insulin levels at 16 weeks of age. n=12 mice for WT-RPTC$^{CB1R+/+}$, n=17 mice for WT-RPTC$^{CB1R-/-}$, n=17 mice for Akita-RPTC$^{CB1R+/+}$, and n=24 mice for Akita-RPTC$^{CB1R-/-}$ (*P<0.0001, $^{\#}$P<0.0001).

(j) Representative insulin staining of pancreatic beta islets in 16-week-old mice.

(k) Pancreas-to-body weight ratio at 16 weeks of age. n=18 mice for WT-RPTC$^{CB1R+/+}$, n=19 mice for WT-RPTC$^{CB1R-/-}$, n=22 mice for Akita-RPTC$^{CB1R+/+}$, and n=33 mice for Akita-RPTC$^{CB1R-/-}$.

The data in C, D, H, I, and K were presented as the means ± SEMs and analyzed by One-way ANOVA followed by one-sided Tukey test. *P<0.05 relative to the corresponding WT-RPTC$^{CB1R+/+}$ control group, $^{\#}$P<0.05 relative to the corresponding WT-RPTC$^{CB1R-/-}$ group, $^{\&}$P<0.05 relative to the corresponding Akita-RPTC$^{CB1R+/+}$ group.

Source data are provided as a Supplementary Source Data file.

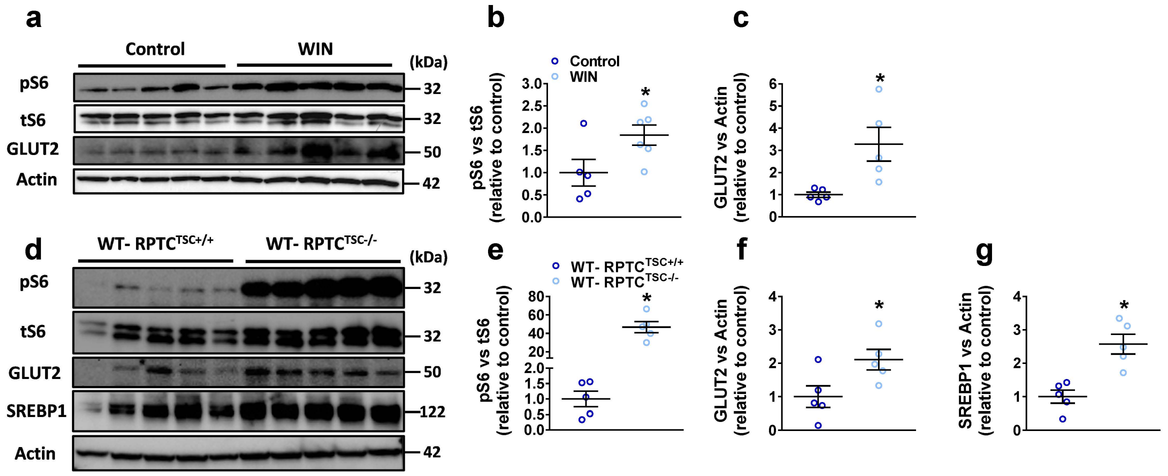

Supplementary Fig 2. S6, GLUT2 and SREBP1 phosphorylation in kidneys of WIN 55,212-injected or WT-RPTC$^{TSC-/-}$ mice, related to Fig 2.

(a) Representative immunoblots for pS6 and GLUT2 in cortical kidney lysates from vehicle- and WIN 55,212- (3 mg/kg) injected mice. n=5 mice per group.

(b-c) Immunoblotting quantification of kidney pS6 and GLUT2 protein levels in cortical kidney lysates from vehicle- and WIN 55,212- (3 mg/kg) injected mice. For **b**, n=5 mice for Control and n=6 mice for WIN. For **c**, n=5 mice per group (*P<0.0472).

(d-g) Immunoblotting quantification of kidney pS6, GLUT2 and SREBP1 protein levels in cortical kidney lysates from WT-RPTC$^{TSC+/+}$ and WT-RPTC$^{TSC-/-}$ mice. n=5 mice per group (*P<0.0385).

Data represent the mean ± SEM and were analyzed by Unpaired Two-tailed Student's t-test.

Source data are provided as a Supplementary Source Data file.

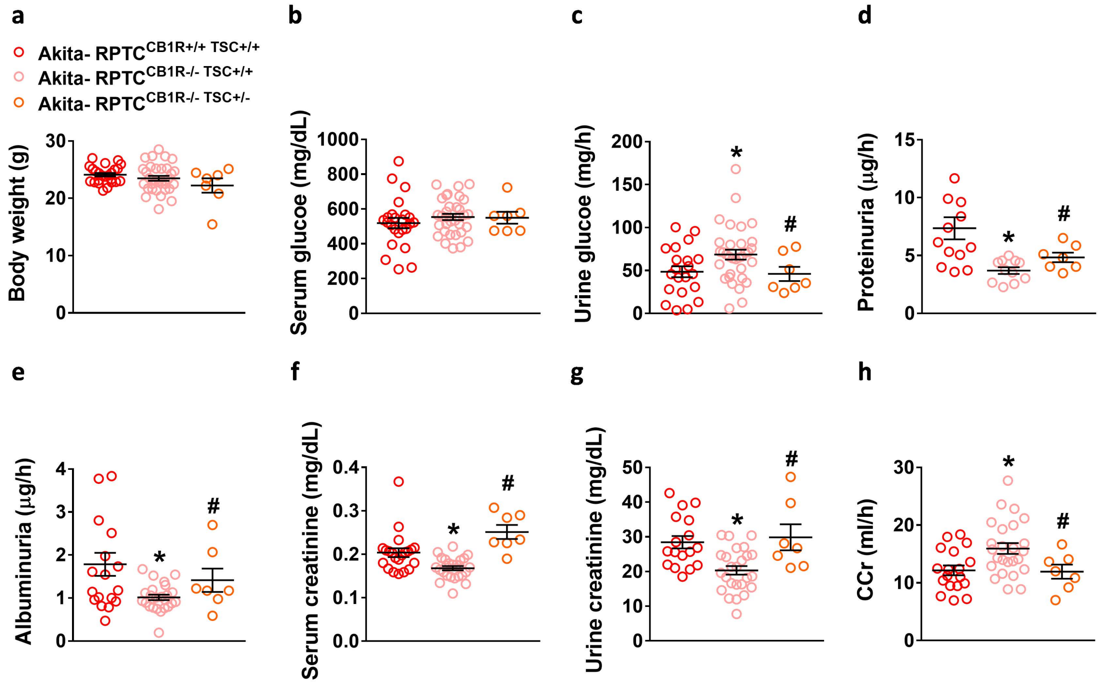

Supplementary Fig 3. Constant RPTC-mTORC1 activation in Akita-RPTC-CB$_1$R$^{-/-}$ mice abolishes the beneficial effects of CB$_1$R deletion on kidney function, related to Fig 2.

(a) Body weight at 16 weeks of age. n=24 mice for Akita-RPTC$^{CB1R+/+TSC+/+}$, n=33 mice for Akita-RPTC$^{CB1R-/-TSC+/+}$, and n=7 mice for Akita-RPTC$^{CB1R-/-TSC+/-}$.

(b) Serum glucose levels at 16 weeks of age. n=24 mice for Akita-RPTC$^{CB1R+/+TSC+/+}$, n=33 mice for Akita-RPTC$^{CB1R-/-TSC+/+}$, and n=7 mice for Akita-RPTC$^{CB1R-/-TSC+/-}$.

(c) Urine glucose levels at 16 weeks of age. n=21 mice for Akita-RPTC$^{CB1R+/+TSC+/+}$, n=33 mice for Akita-RPTC$^{CB1R-/-TSC+/+}$, and n=7 mice for Akita-RPTC$^{CB1R-/-TSC+/-}$ (*P=0.0268, $^{\#}$P=0.0929).

(d) Proteinuria at 16 weeks of age. n=13 mice for Akita-RPTC$^{CB1R+/+TSC+/+}$, n=11 mice for Akita-RPTC$^{CB1R-/-TSC+/+}$, and n=7 mice for Akita-RPTC$^{CB1R-/-TSC+/-}$ (*P=0.0027, $^{\#}$P=0.0325).

(e) Albuminuria at 16 weeks of age. n=17 mice for Akita-RPTC$^{CB1R+/+TSC+/+}$, n=25 mice for Akita-RPTC$^{CB1R-/-TSC+/+}$, and n=7 mice for Akita-RPTC$^{CB1R-/-TSC+/-}$ (*P=0.0024, $^{\#}$P=0.0366).

(f) Serum creatinine levels at 16 weeks of age. n=17 mice for Akita-RPTC$^{CB1R+/+TSC+/+}$, n=25 mice for Akita-RPTC$^{CB1R-/-TSC+/+}$, and n=7 mice for Akita-RPTC$^{CB1R-/-TSC+/-}$ (*P=0.0017, $^{\#}$P<0.0001).

(g) Urine creatinine levels at 16 weeks of age. n=17 mice for Akita-RPTC$^{CB1R+/+TSC+/+}$, n=25 mice for Akita-RPTC$^{CB1R-/-TSC+/+}$, and n=7 mice for Akita-RPTC$^{CB1R-/-TSC+/-}$ (*P=0.0004, $^{\#}$P=0.0039).

(h) Creatinine clearance (CCr) at 16 weeks of age. n=17 mice for Akita-RPTC$^{CB1R+/+TSC+/+}$, n=25 mice for Akita-RPTC$^{CB1R-/-TSC+/+}$, and n=7 mice for Akita-RPTC$^{CB1R-/-TSC+/-}$ (*P=0.0067, $^{\#}$ P=0.0438).

Data represent the mean ± SEM and were analyzed by One-way ANOVA followed by Tukey test (one-sided). *P<0.05 relative to the Akita-RPTC$^{CB1R+/+TSC+/+}$mice; $^{\#}$P<0.05 relative to Akita-RPTC$^{CB1R-/-TSC+/+}$ mice.

Source data are provided as a Supplementary Source Data file.

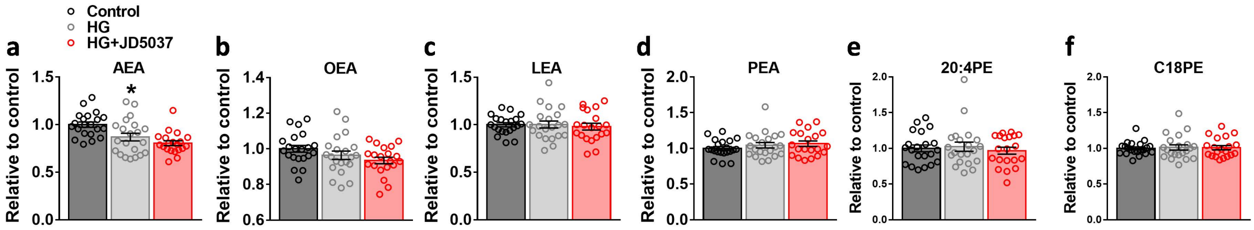

Supplementary Fig 4. Endocannabinoid content in primary hRPTCs, related to Fig 2.

(a-f) LC-MS/MS quantification of endocannabinoids in primary hRPTCs treated with or without HG (30 mM) in the presence or absence of JD5037 (100 nM) for 1 h. n=21 for Control and HG groups and n=20 for HG+JD5037 group (*P=0.0107).

Data represent the mean ± SEM; they were analyzed by One-way ANOVA followed by Tukey test (one-sided). AEA, Anandamide; OEA, Oleoylethanolamine; LEA, Linoleoyl ethanolamide; PEA, Palmitoylethanolamide; 20:4PE, 1, 2-diarachidonoyl-sn-glycero-3-phosphoethanolamine; C18PE, 1-(1Z-octadecenyl)-2-arachidonoyl-sn-glycero-3-phosphoethanolamine.

Source data are provided as a Supplementary Source Data file.

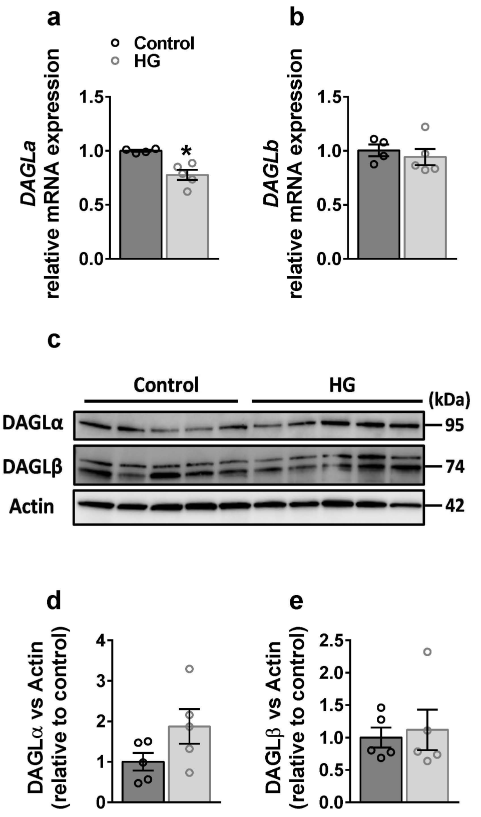

Supplementary Fig 5. DAGL expression in HG-treated primary hRPTCs, related to Fig 2.

(a, b) qPCR analysis of DAGLa and DAGLb mRNA expression levels in primary hRPTCs treated with HG (30 mM) for 3 h. n=5 biological replicates per group (*P=0.0041).

(c-e) Immunoblotting quantification of DAGLα and DAGLβ protein levels in primary hRPTCs treated with HG (30 mM) for 24 h. n=5 biological replicates per group.

Data represent the mean ± SEM; they were analyzed by Unpaired Two-tailed Student's t-test. Source data are provided as a Supplementary Source Data file.

a

P1

\>3 dna:chromosome chromosome:GRCh38:3:170996341:171029647:-1

```
CTCGAGAGCCTCCAGCAAGTGCTTCTTGACATATATGAAGCAATAATAATAACAACCGCTATTTGCTAAGTACTTAT
TATATGTTGAGATATGCCAAACACTTTCACATATTATCTCATTTAATCCACTCAACAAAACAAAATGTGTGTTATTT
CTATTTTATCAGGCAAAAATAAAAAACTAATCAGAGAAAAGTCACAGTGAGCCACTTGCTTAAGGTTATACTCCCCA
GTAAAATGTTGAGATTCAAACCCAAGTCCCTAACAATCCAAAGCTCATCCAGGTAACCATTCAAGTCAACAATGGTC
TCCGGCCACACAGCTCAGCTCTAAACAGAAACACCACAGCACTAATTATCTGTGGAGCAGAGTGCAGTGTGCCTCCC
ATGCTCCACAGCACAGCACGGCAGCTCAGCATATCTCATCTTCTACAAAAGACTCCAAAGATTTCTCTTTTCACCAG
CTCCCAATTACTGCCACATAACACATGCCTTGAAATGTGATTGCCTCTGGTTTGTAACTTATGCCTAAGGGACCTGC
TCCCATTTTCTTTCCTAGTGGAACAAAGGTATTGAAGCCACAGGTTGCTGAGGCAAAGCACTTATTGATTAGATTCC
CATCAATATTCAGCTGCCGCTGAGAAGATTAGACTTGGACTCTCAGGTCTGGGTAGCCCAACTCCTCCCTCTCCTTG
CTCCTCCTCCTGCAATGCATAACTAGGCCTAGGCAGAGCTGCGAATAAACAGGCAGGAGCTAGTCAGGTGCATGTGC
CACACTCACACAAGACCTGGAATTGACAGGACTCCCAACTAGTACAATGACAGAAGATAAGGTAAAGCAGCATTATA
TTCATTTCAAGTCTGGTTTTCAGGAGATAGTTAGGGGAATACAGTCAAGTCATACAGAGGATTTGGGGGAGGTTGGG
TGTAGCTACCACAGATCAAAATTAATTCTCTTTACTTTGCCAATTGAAGAACTGTTTCTTATTCAGTAATTGGGGTT
TTAAGGATTTTTGCTCTGTTAACTGACTAGTCTGCAAGCTT
```

1042bp

XhoI-CTCGAG

HindIII- AAGCTT

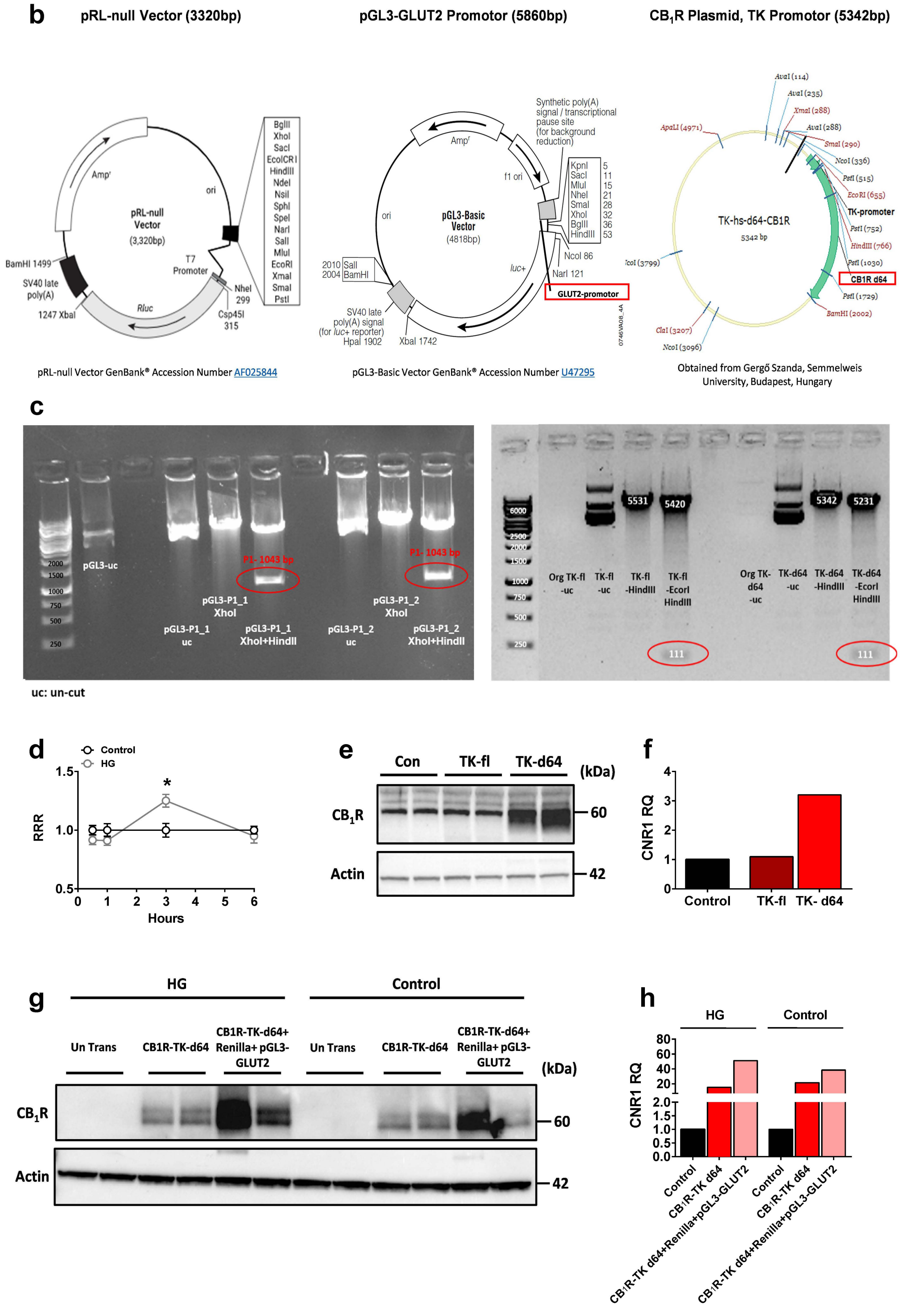

Supplementary Fig 6. Establishing a luminescence-reporting assay for human GLUT2 promoter transcription, related to Fig 4.

(a) Human GLUT2 promoter sequence, containing restriction enzyme sequences at the ends to enable its cloning into the pGL3 plasmid.

(b) Plasmid structure of the three plasmids used in the transfected HEK293 cells.

(c) Plasmid validation using restriction enzymes to ensure that the correct insert was incorporated. Right panel: pGL3-GLUT2 promoter plasmid, left panel: CB_1R-TK plasmid.

(d) Dual-Glo Luciferase assay time course for pGL3-GLUT2 transfected HEK293 cells, treated with or without HG (30 mM glucose) for 6 h. n=8 wells per group (3h, *P=0.0088).

(e, f) Immunoblotting analysis and quantification of CB_1R protein in CB_1R-TK-fl or CB_1R-TK-d64 plasmid-transfected HEK293 cells. n=2 per group, fl- full length CB_1R, d64- short CB_1R version.

(g, h) Immunoblotting analysis and quantification of CB_1R protein in non-transfected, single CB_1R-TK- d64 transfected or triple CB_1R-TK- d64, pGL3-GLUT2, and pRL-null transfected HEK293 cells, treated with or without HG (30 mM glucose) for 3 h. n=2 per group.

The data in d was presented as the mean ± SEM and analyzed by Unpaired Two-tailed Student's t-test.

Source data are provided as a Supplementary Source Data file.

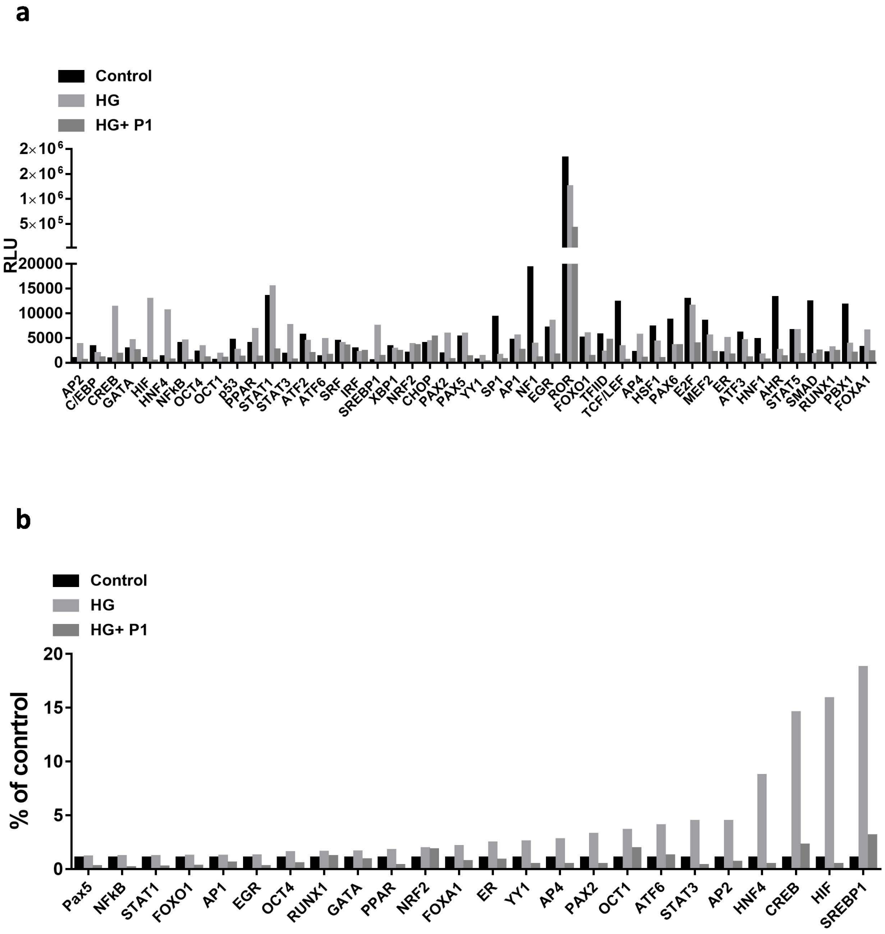

Supplementary Fig 7. Promoter-binding transcription-factor (TF) profiling assay for GLUT2 TF candidates, related to Fig 4.

(a) Promoter-binding transcription-factor profiling assay for 47 candidate TFs in nuclear extracts (NEs) from hRPTCs treated with or without HG (30 mM of glucose) for 3 h in the presence or absence of hGLUT2 promoter (P1).

(b) TFs, enriched in the NEs of hRPTCs, treated with HG (30 mM) for 3 h and competitively inhibited by hGLUT2 promoter (P1).

Source data are provided as a Supplementary Source Data file.

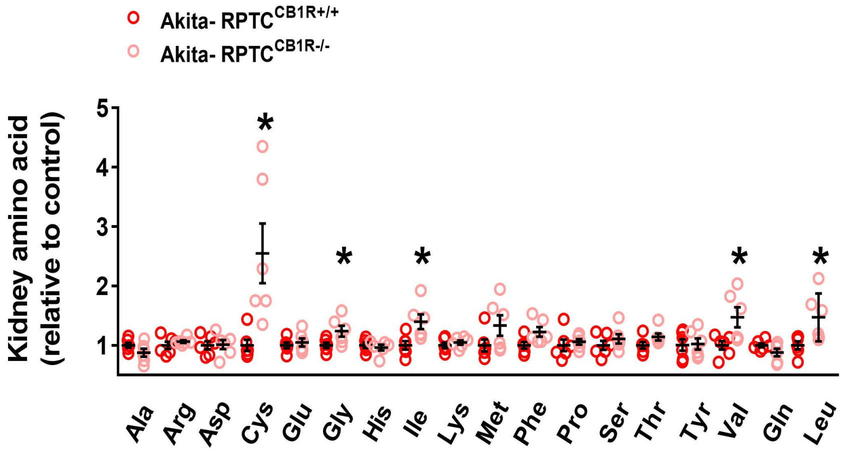

Supplementary Fig 8. Kidney BCAAs in Akita-RPTC$^{CB1R-/-}$ diabetic mice, related to Fig 5.

LC-MS/MS quantification of amino acids in kidney lysates from Akita-RPTC$^{CB1R+/+}$ and Akita-RPTC$^{CB1R-/-}$ diabetic mice. n=6 mice per group (*P<0.0400).

Data represent the mean ± SEM and were analyzed by Unpaired Two-tailed Student's t-test.

Source data are provided as a Supplementary Source Data file.

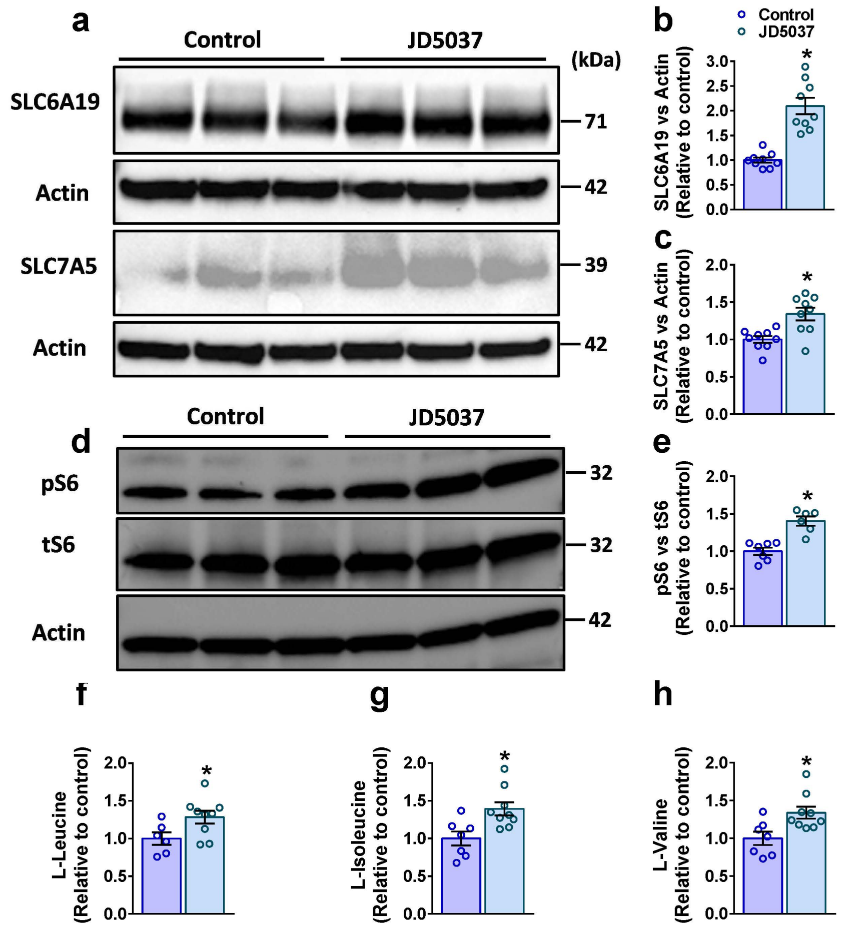

Supplementary Fig 9. CB_1R blockade enhances amino acid transport in hRPTCs, related to Fig 5.

(a) Representative immunoblots for SLC6A19 and SLC7A5 in hRPTCs treated with JD5037 (100 nM) for 24 h. n=3 biological replicates per group.

(b, c) Immunoblotting quantification of SLC6A19 and SLC7A5 protein levels in primary hRPTCs treated with JD5037 (100 nM) for 24 h. n=9 per group (*P<0.0026).

(d) Representative immunoblots for pS6 in hRPTCs treated with JD5037 (100 nM) for 24 h. n=3 per group.

(e) Immunoblotting quantification of pS6 protein levels of primary hRPTCs treated with JD5037 (100 nM) for 24 h. n=7 for Control and n=6 for JD5037 (*P<0.0003).

(**f-h**) LC-MS/MS quantification of amino acid uptake in hRPTCs treated with L-Leucine, L-isoleucine, and L-valine for 3 h in the presence or absence of JD5037 (100 nM). For **f**, n=6 for

Control and n=9 for JD5037 (*P=0.0408). For **g**, n=7 for Control and n=9 for JD5037 (*P=0.0089). For **h**, n=7 for Control and n=9 for JD5037 (*P=0.0125).

Data represent the mean ± SEM analyzed by Unpaired Two-tailed Student's t-test.

Source data are provided as a Supplementary Source Data file.

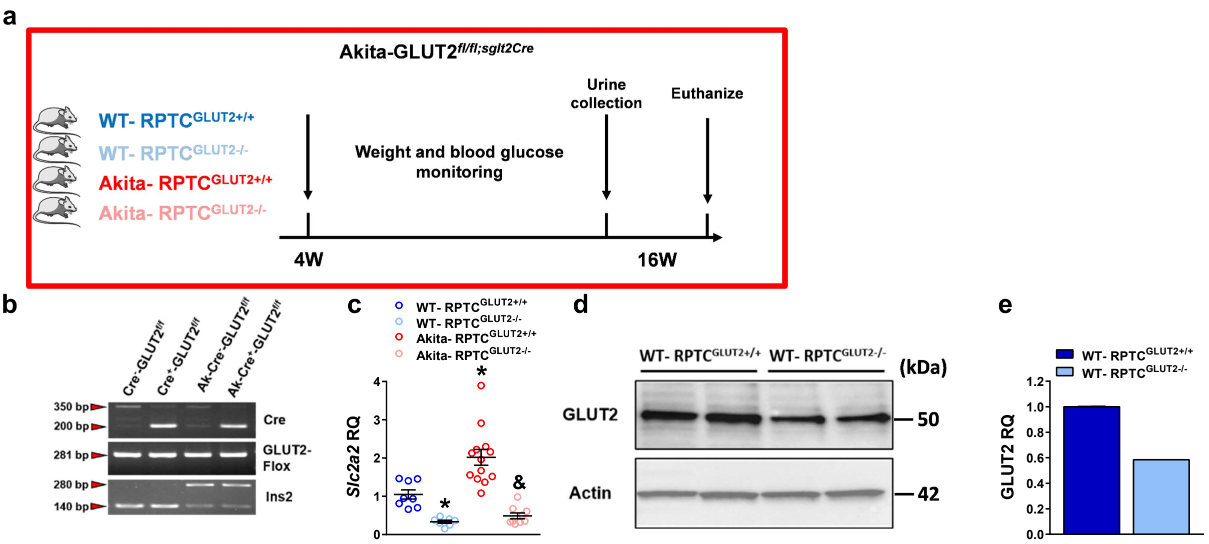

Supplementary Fig 10. Generation and Characterization of Akita-RPTC-GLUT2 null mice, related to Fig 7.

(a) Scheme of the experimental paradigm using WT and Akita mice, with or without reduced expression of GLUT2 in RPTCs. The illustration of the mice was prepared using Servier Medical Art website (smart.servier.com).

(b) Typical genotyping of each experimental group for Cre recombinase, GLUT2-flox, and Ins2.

(c) qPCR analysis of kidney *Slc2a2* (GLUT2) mRNA expression levels in each of the experimental groups. n=8 mice for WT-RPTC$^{GLUT2+/+}$, n=7 mice for WT-RPTC$^{GLUT2-/-}$, n=13 mice for Akita-RPTC$^{GLUT2+/+}$, n=9 mice for Akita-RPTC$^{GLUT2-/-}$ (*P<0.0001, $^{\&}$P<0.0001). The mRNA expression level of target genes was normalized to that of *Ubc* (ubiquitin C).

(d, e) Immunoblotting analysis and quantification of kidney GLUT2 in RPTCs isolated from mouse kidneys. n=2 mice per group.

The data are presented as the mean ± SEM and analyzed by One-way ANOVA followed by Tukey test (one-sided). In c, *P<0.05 relative to the corresponding WT-RPTC$^{GLUT2+/+}$ control group, $^{\&}$P<0.05 relative to the corresponding Akita-RPTC$^{GLUT2+/+}$ group.

Source data are provided as a Supplementary Source Data file.

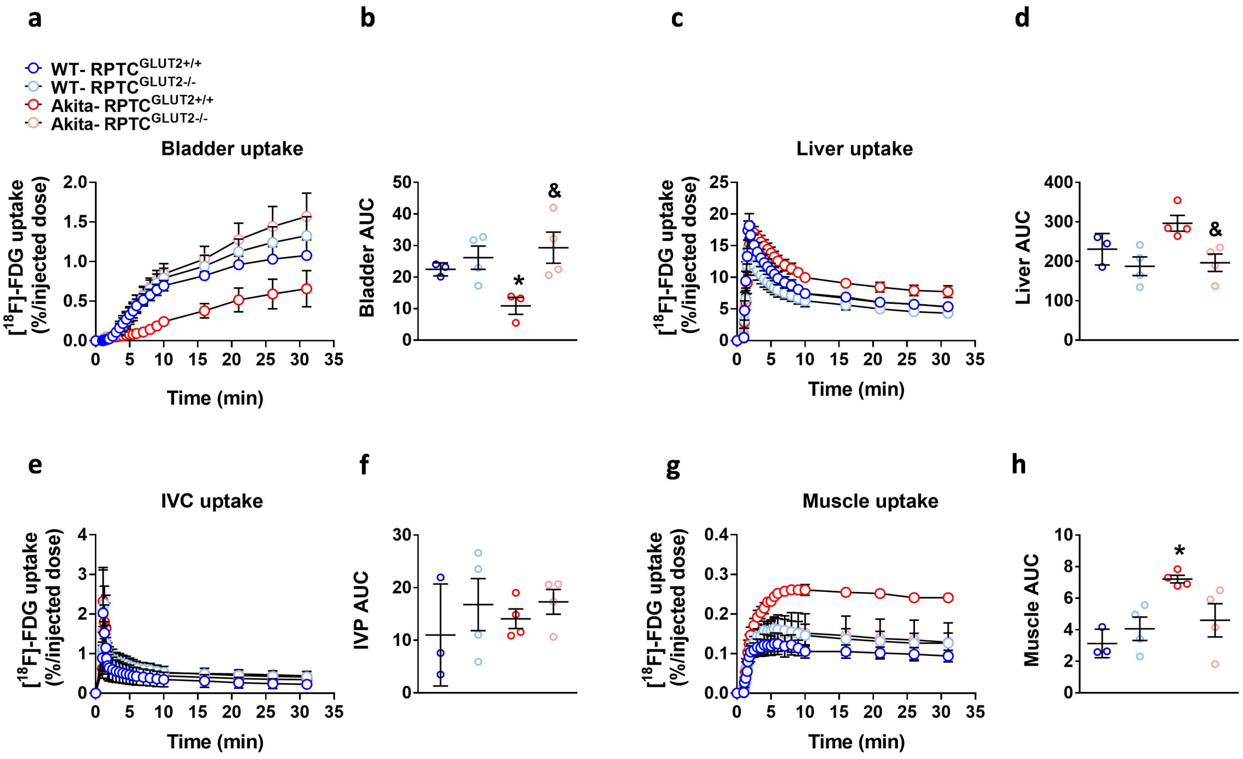

Supplementary Fig 11. Tissue uptake of $[^{18}F]$-FDG in WT and Akita RPTC-GLUT2$^{-/-}$ mice, related to Fig 7.

Bladder **(a, b)**, liver **(c, d)**, inferior vena cava (IVC; **e, f**), and muscle **(g, h)** uptake of $[^{18}F]$-FDG using PET-MRI analysis. For **a** and **b**, n=3 mice for WT-RPTC$^{GLUT2+/+}$ and Akita-RPTC$^{GLUT2+/+}$, n=4 mice for WT-RPTC^{GLUT2} and Akita-RPTC$^{GLUT2-/-}$ (*P=0.0169, $^{\&}$P=0.0318). For **c-h**, n=3 mice for WT-RPTC$^{GLUT2+/+}$, n=4 mice for WT-RPTC^{GLUT2}, Akita-RPTC$^{GLUT2+/+}$ and Akita-RPTC$^{GLUT2-/-}$ (*P=0.0005, $^{\&}$P=0.0151).

The data are presented as the mean ± SEM and were analyzed by One-way ANOVA followed by Tukey test (one-sided). *P<0.05 relative to the corresponding WT-RPTC$^{GLUT2+/+}$ control group, $^{\&}$P<0.05 relative to the corresponding Akita-RPTC$^{GLUT2+/+}$ group.

Source data are provided as a Supplementary Source Data file.

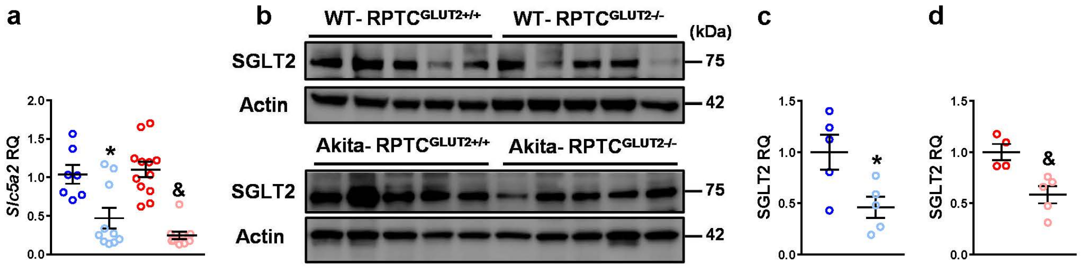

Supplementary Fig 12. SGLT2 expression in the kidney lysate of WT and Akita RPTC-GLUT2$^{-/-}$ mice, related to Fig 7.

(a) qPCR analysis of kidney *Slc5a2* (SGLT2) mRNA expression levels in each of the experimental groups. n=7 mice for WT-RPTC$^{GLUT2+/+}$, n=10 mice for WT-RPTC$^{GLUT2-/-}$, n=12 mice for Akita-RPTC$^{GLUT2+/+}$, n=10 mice for Akita-RPTC$^{GLUT2-/-}$ (*P=0.0087, $^{\&}$P<0.0001). The mRNA expression level of the target gene was normalized to that of *Ubc* (ubiquitin C).

(b-d) Immunoblotting analysis and quantification of kidney SGLT2 in cortical kidney lysates. n=5 mice for WT-RPTC$^{GLUT2+/+}$, WT-RPTC$^{GLUT2-/-}$ and Akita-RPTC$^{GLUT2-/-}$, n=4 mice for Akita-RPTC$^{GLUT2+/+}$ (*P=0.0283, $^{\&}$P=0.0093).

The data are presented as the mean ± SEM and were analyzed by Unpaired Two-tailed Student's t-test or One-way ANOVA followed by Tukey test (one-sided). *P<0.05 relative to the corresponding WT-RPTC$^{GLUT2+/+}$ control group, $^{\&}$P<0.05 relative to the corresponding Akita-RPTC$^{GLUT2+/+}$ group.

Source data are provided as a Supplementary Source Data file.

Supplementary Table 1. Mouse primers

Gene	Forward primer (5'-3')	Reverse primer (5'-3')
Atf6	TCGCCTTTTAGTCCGGTTCTT	GGCTCCATAGGTCTGACTCC
Bcat1	GTCTGCCCAGTCTCTGATATTC	ACTCTCCACCCTTCCATACT
Bcat2	GCTGATGGTGGAGTGGAATAA	CTCAAAGAGCTGCAGAGAGTAG
Bckdha	CGGAAGCAGTCACGAAAGAA	TCCTGGTACACATCGGAGAA
Clu	AGAGCTCACCCTTCTACTTCTG	TCCACCTTCTCTTAAGAAATCAAC
Cnr1	AAGTCGATCTTAGACGGCCTT	TCCTAATTTGGATGCCATGTCTC
Col1	TTCTCCTGGCAAAGACGGACTCAA	GGAAGCTGAAGTCATAACCGCCA
Col3	ACAGCAAATTCACTTACACAGTTC	CTCATTGCCTTGCGTGTTT
Creb	CATTGCCCCTGGAGTTGTTATG	TTCTCTTGCTGCCTCCCTGTT
Faah	GTATCGCCAGTCCGTCATTG	GCCTATACCCTTTTTCATGCCC
Fabp4	GATGCCTTTGTGGGAACCTG	GCCATGCCTGCCACTTTC
Fn1	ATGTGGACCCCTCCTGATAGT	GCCCAGTGATTTCAGCAAAGG
Hif1a	TTGCTTTGATGTGGATAGCGATA	CATACTTGGAGGGCTTGGAGAAT
Hnf4a	AAATGTGCAGGTGTTGACCA	CACGCTCCTCCTGAAGAATC
Ip-10	GGATGGCTGTCCTAGCTCTG	TGAGCTAGGGAGGACAAGGA
Il-18	GACTCTTGCGTCAACTTCAAGG	CAGGCTGTCTTTTGTCAACGA
Kim1	TGTCGAGTGGAGATTCCTGGATGGT	GGTCTTCCTGTAGCTGTGGGCC
Lcn2	TTTCACCCGCTTTGCCAAGT	GTCTCTGCGCATCCCAGTCA
Lrp2	AAAATGGAAACGGGGTGACTT	GGCTGCATACATTGGGTTTTCA
Mcp1	GCATTAGCTTCAGATTTA	TTAAAAACCTGGATCGGAACCAA
Mgll	ACCATGCTGTGATGCTCTCTG	CAAACGCCTCGGGGATAACC
Slc2a2	TCAGAAGACAAGATCACCGGA	GCTGGTGTGACTGTAAGTGGG
Slc3a1	AGGCACTCTCTCTGGGCATA	GGGAGTGTGAACAGGAGCAT
Slc5a2	ATGCGCTCTTCGTGGTGCTG	ACCAAAGCGCTTGCGGAGGT
Slc6a19	GGAGTGTGCTGTATGTGTGTAT	TCAAGCCACGGATGAGAAAG
Slc7a5	CCTACGGAGGATGGAACTATCT	TGACAATGGGCAAGGAGATG
Srebp1c	GGAGCCATGGATTGCACATT	GCTTCCAGAGAGGAGGCCAG
Stat3	AGGAGTCTAACAACGGCAGC	ACAGGATTGATGCCCAAGCA
Tgfb	GCGGACTACTATGCTAAAGAGG	GTAGAGTTCCACATGTTGCTCC
Ubc	CAGCCGTATATCTTCCCAGAC	CTCAGAGGGATGCCAGTAATC

Tnf	QT00104006 QuantiTect Primer Assays (Qiagen, Germany)
Cst3	QT00113155 QuantiTect Primer Assays (Qiagen, Germany)

Supplementary Table 2. Human primers

Gene	Forward primer (5'-3')	Reverse primer (5'-3')
BCAT1	AGCCCTGCTCTTTGTACTCTT	CCAGGCTCTTACATACTTGGGA
BCAT2	GCTCAACATGGACCGGATG	CCGCACATAGAGGCTGGTG
BCKDH	TGGCTAGATCTCACCCC	AGAGAATGCGGTCCATGGTG
DAGLa	CCATCTTCCTCTTTCTCCT	CTCGTGCGGGTTATAGAC
DAGLb	TCAGGTGCTACGCCTTCTC	TCACACTGAGCCTGGGAATC
SLC2A2	TGGGCTGAGGAAGAGACTGT	AGAGACTGAAGGATGGCTCG
SLC3A1	CAGGAGCCCGACTTCAAGG	GAGGGCAATGATGGCTATGGT
SLC6A19	CAGCAACAACTGCGAGAAGG	CAATGACGGAGTAGACCACGAT
SLC7A5	CTTCGGCTCTGTCAATGGGT	TTCACCTTGATGGGACGCTC
GAPDG	AATCCCATCACCATCTTCCA	TGGACTCCACGACGTACTCA
RPLP0	CCAACTACTTCCTTAAGATCATCCAA	ACATGCGGATCTGCTGCTGCA

Supplementary Table 3. Candidate TFs suggested by TFBIND software for GLUT2 promotor (related to Fig 4F, G and Supplementary Fig 4)

**1**	AP2	**38**	AML1	**75**	AHR	**112**	PADS
**2**	AP4	**39**	GATA2	**76**	HSF2	**113**	ARP1
**3**	CP2	**40**	CEBPB	**77**	HEN1	**114**	NFE2
**4**	E47	**41**	CEBPA	**78**	EGR2	**115**	CLOX
**5**	ARNT	**42**	CEBP	**79**	EGR3	**116**	STAT
**6**	CMYB	**43**	HLF	**80**	NGFIC	**117**	TST1
**7**	CETS1P54	**44**	MEF2	**81**	RSRFC4	**118**	BARBIE
**8**	NRF2	**45**	LYF1	**82**	CHOP	**119**	IK1
**9**	MYCMAX	**46**	MYOGNF1	**83**	SP1		
**10**	MAX	**47**	EVI1	**84**	PPARA		
**11**	USF	**48**	HFH1	**85**	XBP1		
**12**	MYOD	**49**	P53	**86**	IRF1		
**13**	NMYC	**50**	IK3	**87**	POLY_C		
**14**	TAXCREB	**51**	TH1E47	**88**	COUP		
**15**	LMO2COM	**52**	E2F	**89**	OLF1		
**16**	NKX25	**53**	CAP	**90**	IK2		
**17**	ER	**54**	E4BP4	**91**	DELTAEF1		
**18**	XFD3	**55**	AHRARNT	**92**	ZID		
**19**	OCT1	**56**	T3R	**93**	ATF		
**20**	CREB	**57**	NFKAPPAB	**94**	AP1		
**21**	HOX13	**58**	CREBP1CJUN	**95**	S8		
**22**	NFY	**59**	ELK1	**96**	OCT		
**23**	PBX1	**60**	GATA3	**97**	NFKB		
**24**	SOX5	**61**	GATA1	**98**	NF1		
**25**	CDP	**62**	AP1FJ	**99**	IRF2		
**26**	YY1	**63**	HNF1	**100**	PAX6		
**27**	PAX2	**64**	CREL	**101**	VBP		
**28**	CREBP1	**65**	TAF	**102**	RREB1		
**29**	ATF2	**66**	MZF1	**103**	ISRE		
**30**	RORA	**67**	VJUN	**104**	XFD2		
**31**	RORA2	**68**	GFI1	**105**	HSF1		
**32**	SRF	**69**	HNF4	**106**	P300		
**33**	TATA	**70**	HNF3B	**107**	EGR1		
**34**	CAAT	**71**	SREBP1	**108**	PAX5		
**35**	RFX1	**72**	COMP1	**109**	MIF1		
**36**	CDXA	**73**	CDPCR1	**110**	XFD1		
**37**	VMYB	**74**	CDPCR3HD	**111**	HFH2		

Supplementary Table 4. Potential candidate GLUT2 TFs (related to Fig 4F, G and Supplementary Fig 4)

**1**	AP2- Transcription factor AP-2 alpha
**2**	C/EBP- CCAAT/enhancer binding protein (C/EBP),alpha
**3**	CREB- cAMP responsive element binding protein 1
**4**	GATA- GATA transcription factor
**5**	HIF- Hypoxia inducible factor
**6**	HNF4- Hepatocyte nuclear factor 4
**7**	NFkB- Nuclear factor of kappa light polypeptide
**8**	OCT4- POU class 5 homeobox 1
**9**	OCT1- POU domain, class 2, transcription factor
**10**	p53- Tumor protein p53
**11**	PPAR- Peroxisome proliferator-activated receptor
**12**	STAT1- Signal transducer and activator of transcription 1
**13**	STAT3- Signal transducer and activator of transcription 3
**14**	ATF2- Activating transcription factor 2
**15**	ATF6- Activating transcription factor 6
**16**	SRF- Serum response factor
**17**	IRF- Interferon regulatory factor
**18**	SREBP1- sterol regulatory element binding transcription factor 1
**19**	XBP1- X-box binding protein 1
**20**	NRF2-related antioxidant responsive
**21**	CHOP- DNA damage inducible transcript 3 (DDIT3)
**22**	PAX2- Pair box-2 protein
**23**	PAX5- Paired box 5
**24**	YY1- YY1 transcription factor
**25**	SP1- SP1 transcription factor
**26**	AP1- Jun proto-oncogene, AP-1 transcription factor subunit
**27**	NF-1 Nuclear factor 1
**28**	EGR- Early growth response
**29**	ROR- Retinoic acid receptor-related orphan
**30**	FOXO1- (FKHR) FOXbox O1
**31**	TFIID- TATA box binding protein
**32**	TCF/LEF- (HNF4a) hepatocyte nuclear factor 4 alpha
**33**	AP4- Transcription factor AP-4 (activating enhancer binding protein 4)
**34**	HSF1- heat shock transcription factor 1
**35**	PAX6- paired box 6
**36**	E2F- E2F transcription factor 1
**37**	MEF2- myocyte enhancer factor 2A
**38**	ER- Estrogen receptors (ERs)
**39**	ATF3 Activating transcription factor 3
**40**	HNF1- Hepatocyte nuclear factor 1 homeobox A
**41**	AHR- aryl hydrocarbon receptor

42 STAT5- Signal transducer and activator of transcription 5
43 SMAD- SMAD family member
44 RUNX1- RUNX family transcription factor 1
45 PBX1- PBX homeobox 1
46 FOXA1- forkhead box A1

Supplementary Table 5. MRM transitions for eCBs measurements in ESI+ and ESI-. (related to Fig 2R-T and Supplementary Fig 2). Tam Lab, Hebrew University and Gertsch Lab, University of Bern, Switzerland

Analyte	Molecular ion $[M+H]^+$ $[M-H]^-$ for AA [m/z]	Fragment [m/z]	DP [volts]	CE [volts]	CXP [volts]	
2-AG	379.2	287.1 (quantifier)	70	19	14	Tam Lab
		91 (qualifier)	70	67	10	
AEA	348.2	287.1 (quantifier)	26	13	16	
		62 (qualifier)	26	13	8	
PEA	300.3	283.2 (quantifier)	130	19	24	
		62 (qualifier)	130	17	8	
AA	305.3	91 (quantifier)	1	49	10	
		287.1 (qualifier)	1	13	22	
OEA	326.3	61.9 (quantifier)	146	21	24	
		309.1 (qualifier)	146	21	42	
d_4-AEA	352.3	287.1 (quantifier)	66	15	20	
		66 (qualifier)	66	21	8	
2-AG	379.1	287.2 (quantifier)	141	21	12	Gertsch Lab
		93.0 (qualifier)	126	49	12	
AEA	348.2	62.0 (quantifier)	81	17	8	
		91.0 (qualifier)	81	61	12	
LEA	324.2	66.9 (quantifier)	91	63	10	
		306.2 (qualifier)	101	19	12	
PEA	300.3	62.0 (quantifier)	105	18	8	
		282.2 (qualifier)	101	27	12	
OEA	326.4	309.2 (quantifier)	116	21	12	
		69.0 (qualifier)	96	49	10	
SAG	662.4	341.1 (quantifier)	111	25	14	
	667.4	327.1 (qualifier)	76	41	14	
AA	303	259.1 (quantifier)	-135	-18	-33	
		59.0 (qualifier)	-130	-26	-9	
d_5-2-AG	384.1	287.2	116	21	12	
d_4-AEA	352.2	66.0	86	19	8	
d_4-LEA	328.1	66.0	81	19	10	
d_4-PEA	305.0	62.0	105	40	8	
d_4-OEA	330.3	66.0	101	21	10	
d_8-AA	311.1	59.0	-120	-26	-9	
d_8-SAG	670.4	341.2	91	27	14	

2-Arachidonoylglycerol, 2-AG; Anandamide, AEA; Linoleoyl ethanolamide, LEA; Palmitoylethanolamide, PEA; Oleoylethanolamine, OEA; 1-stearoyl-2-arachidonoyl-sn-glycerol, SAG; Arachidonic acid, AA.

Supplementary Table 6. Optimized MRM transitions for BCAAs (related to Fig 5Q and Supplementary Fig 5)

Analyte	Molecular ion $[M+H]^+$ (m/z)	Fragment (m/z)	DP (volts)	CE (volts)	CXP (volts)	Retention time (min)
Alanine (Ala)	90.0	44.0 (quantifier)	61	13	20	9.00
		42.0 (qualifier)	61	55	38	
Arginine (Arg)	175.2	70 (quantifier)	36	25	8	15.90
		116.1 (qualifier)	36	19	12	
Aspartic Acid (Asp)	134.1	88.0 (quantifier)	56	13	10	9.45
		73.9 (qualifier)	56	17	8	
Cystine (Cys)	241.1	151.9 (quantifier)	36	17	18	11.00
		119.9 (qualifier)	36	25	18	
Glutamic acid (Glu)	148.0	102.0 (quantifier)	46	19	12	9.30
		84.1 (qualifier)	46	21	8	
Glutamine (Gln)	147.1	130 (quantifier)	36	13	14	9.40
		84 (qualifier)	36	23	8	
Glycine (Gly)	76.0	30.0 (quantifier)	36	17	12	9.40
Histidine (His)	156.0	110.0 (quantifier)	56	19	12	13.90
		83.0 (qualifier)	56	31	10	
Isoleucine (Ile)	132.1	86.2 (quantifier)	51	13	10	5.80
		69.0 (qualifier)	51	20	32	
Leucine (Leu)	132.1	86 (quantifier)	51	13	10	5.50
		44 (qualifier)	51	27	4	
Lysine (Lys)	146.9	84.1 (quantifier)	51	23	10	14.30
		130.0 (qualifier)	51	13	14	
Methionine (Met)	150.0	133 (quantifier)	41	11	16	6.45
		104 (qualifier)	41	13	12	
Phenylalanine (Phe)	166.1	120.0 (quantifier)	36	19	12	5.00
		61.0 (qualifier)	36	35	10	
Proline (Pro)	116.1	70 (quantifier)	41	19	10	6.90
		43 (qualifier)	41	39	20	
Serine (Ser)	106.0	60 (quantifier)	46	13	8	9.60
		88 (qualifier)	46	13	12	
Threonine (Thr)	120.1	103 (quantifier)	111	23	12	5.00
		77 (qualifier)	111	33	10	
Tyrosine (Tyr)	182.1	136 (quantifier)	36	17	6	7.10
		165 (qualifier)	36	13	14	
Valine (Val)	118.2	72 (quantifier)	56	13	8	7.10
		55 (qualifier)	56	25	8	
S-(2-Aminoethyl)-L-cysteine hydrochloride	165.3	120 (quantifier)	26	17	14	14.00
		148 (qualifier)	26	11	14	

Supplementary Table 7. Amino acids raw levels (related to Fig 5Q and Supplementary Fig 5)

**Amino Acid**	**WT-RPTC$^{CB1R+/+}$ (µM/mg)**	**WT-RPTC$^{CB1R-/-}$ (µM/mg)**	**Akita-RPTC$^{CB1R+/+}$ (µM/mg)**	**Akita-RPTC$^{CB1R-/-}$ (µM/mg)**
Alanine	2.123±0.128	2.075±0.072	2.154±0.092	1.888±0.146
Arginine	0.092±0.007	0.110±0.002*	0.101±0.006	0.107±0.002
Aspartic Acid	2.377±0.211	2.009±0.082	2.685±0.18	2.727±0.175
Cystine	0.223±0.012	0.504±0.041*	0.392±0.037	1.000±0.197*
Glutamic acid	6.814±0.144	8.482±0.394*	7.918±0.380	8.304±0.540
Glutamine	0.996±0.064	1.166±0.021*	0.836±0.028	0.736±0.053
Glycine	5.794±0.198	7.157±0.165*	5.167±0.231	6.402±0.469*
Histidine	0.150±0.006	0.206±0.003*	0.191±0.008	0.180±0.012
Isoleucine	0.148±0.008	0.178±0.012	0.188±0.013	0.246±0.025*
Leucine	0.272±0.010	0.320±0.017*	0.354±0.024	0.521±0.058*
Lysine	0.083±0.006	0.097±0.002	0.075±0.003	0.079±0.003
Methionine	0.094±0.004	0.112±0.005*	0.086±0.009	0.115±0.015
Phenylalanine	0.116±0.002	0.124±0.008	0.135±0.007	0.159±0.012
Proline	0.153±0.003	0.145±0.009	0.148±0.015	0.157±0.007
Serine	0.337±0.018	0.406±0.018*	0.355±0.027	0.393±0.028
Threonine	0.119±0.003	0.124±0.007	0.141±0.008	0.161±0.008
Tyrosine	0.112±0.004	0.139±0.015	0.115±0.009	0.121±0.009
Valine	0.241±0.007	0.281±0.015*	0.318±0.023	0.469±0.053*

Values presented as the mean ± SEM and analyzed by Student's t-test. *$P<0.05$ relative to the corresponding WT-RPTC$^{CB1R+/+}$ control group or to the corresponding Akita-RPTC$^{CB1R+/+}$ group.
