# Supplementary material for: Opposite physiological and pathological mTORC1-mediated roles of the CB1 receptor in regulating renal tubular function
Source: Nat Commun. 2022 Apr 4;13:1783. doi: 10.1038/s41467-022-29124-8 (PMC8980033; doi:10.1038/s41467-022-29124-8)
Supplement: Supplementary file 3 — Reporting Summary [file 41467_2022_29124_MOESM3_ESM.pdf]

nature research

Corresponding author(s): Joseph Tam

Last updated by author(s): Jan 25, 2022

# Reporting Summary

Nature Research wishes to improve the reproducibility of the work that we publish. This form provides structure for consistency and transparency in reporting. For further information on Nature Research policies, see our Editorial Policies and the Editorial Policy Checklist.

## Statistics

For all statistical analyses, confirm that the following items are present in the figure legend, table legend, main text, or Methods section.

| n/a | Confirmed | |
|---|---|---|
| ☐ | ☒ | The exact sample size (*n*) for each experimental group/condition, given as a discrete number and unit of measurement |
| ☐ | ☒ | A statement on whether measurements were taken from distinct samples or whether the same sample was measured repeatedly |
| ☐ | ☒ | The statistical test(s) used AND whether they are one- or two-sided<br>*Only common tests should be described solely by name; describe more complex techniques in the Methods section.* |
| ☐ | ☒ | A description of all covariates tested |
| ☐ | ☒ | A description of any assumptions or corrections, such as tests of normality and adjustment for multiple comparisons |
| ☐ | ☒ | A full description of the statistical parameters including central tendency (e.g. means) or other basic estimates (e.g. regression coefficient) AND variation (e.g. standard deviation) or associated estimates of uncertainty (e.g. confidence intervals) |
| ☐ | ☒ | For null hypothesis testing, the test statistic (e.g. *F*, *t*, *r*) with confidence intervals, effect sizes, degrees of freedom and *P* value noted<br>*Give P values as exact values whenever suitable.* |
| ☒ | ☐ | For Bayesian analysis, information on the choice of priors and Markov chain Monte Carlo settings |
| ☒ | ☐ | For hierarchical and complex designs, identification of the appropriate level for tests and full reporting of outcomes |
| ☒ | ☐ | Estimates of effect sizes (e.g. Cohen's *d*, Pearson's *r*), indicating how they were calculated |

*Our web collection on statistics for biologists contains articles on many of the points above.*

## Software and code

Policy information about availability of computer code

| | |
|---|---|
| Data collection | ZEN Blue Software (Zeiss microscopy) - Images data collection<br>TFBIND software (https://tfbind.hgc.jp/) - Transcription factors data collection<br>Cobas C-111 chemistry analyzer (Roche, Switzerland) - serum and urine biochemistry data collection<br>ChemiDoc™ Touch Imaging System (Bio-Rad, CA)- Western blot and DNA gels data collection<br>MRS*DRYMAG 7.0T (MR Solutions, Guildford, UK)- PET-MRI images data collection<br>CFX connect ST system (Bio-Rad, CA)- Real time PCR data collection<br>AB Sciex (Framingham, MA, USA) QTRAP® 6500 + mass spectrometer coupled with a Shimadzu (Kyoto, Japan) UHPLC System- LC-MS/MS data collection<br>AB Sciex (Framingham, MA, USA) Triple Quad™ 5500 mass spectrometer coupled to a Shimadzu (Kyoto, Japan) UHPLC System- LC-MS/MS data collection |
| Data analysis | ZEN 2.3 software (Zeiss Microscopy) - Images data analysis<br>Image J version 1.44 software - Images data analysis<br>Image Lab version 6 - Western blots and DNA gels data analysis<br>VivoQuant pre-clinical image post-processing software (Invicro)- PET-MRI images data analysis<br>Bio-Rad CFX Manager software v Maestro 1.1 - Real time qPCR data analysis<br>Analyst software version 1.6 - LC-MS/MS data analysis<br>All statistical analysis were preformed using GraphPad Prism version 6 (GraphPad Software, Inc.) |

For manuscripts utilizing custom algorithms or software that are central to the research but not yet described in published literature, software must be made available to editors and reviewers. We strongly encourage code deposition in a community repository (e.g. GitHub). See the Nature Research guidelines for submitting code & software for further information.

# Data

Policy information about availability of data

All manuscripts must include a data availability statement. This statement should provide the following information, where applicable:

- Accession codes, unique identifiers, or web links for publicly available datasets
- A list of figures that have associated raw data
- A description of any restrictions on data availability

All data that support the findings of this study are available within the article, its Supplementary Information or Source Data files. Primers lists are provided in Supplementary Tables 1 & 2 (in the Supplementary Information file). Uncropped gels are available in the Supplementary Information file. Source data are provided with this paper.

# Field-specific reporting

Please select the one below that is the best fit for your research. If you are not sure, read the appropriate sections before making your selection.

☒ Life sciences ☐ Behavioural & social sciences ☐ Ecological, evolutionary & environmental sciences

For a reference copy of the document with all sections, see nature.com/documents/nr-reporting-summary-flat.pdf

# Life sciences study design

All studies must disclose on these points even when the disclosure is negative.

| | |
|---|---|
| Sample size | The sample size for each treatment group is detailed in the Figure Legends and Materials and Methods section. No explicit power analysis was conducted to pre-determine sample size. It was basically based on our substantial prior experience with similar in vivo studies to detect main effects at a power of 0.80 and alpha of p<0.05. |
| Data exclusions | The number of biological replicates for each experiment is noted in corresponding figure legends. Two mice were excluded from the histological assessment of the kindey (Figure 7p) due to technical issues in the parafin embedding of their kidney samples. |
| Replication | In vivo mouse experiments were performed in a continuance manner, during a long period of time, in which male pups from each mouse strain were collected from every litter, genotyped, and added to its corresponding group. Therefore, each biological replicate in the presented in vivo mouse experiments corresponds to an individual mouse.<br><br>All in vitro experiments were done independently and replicated at least twice. |
| Randomization | Mouse littermates were divided into their experimental groups according to their genotypes as detailed in the methods.<br><br>In the in vitro studies, cells were randomly allocated into experimental groups. |
| Blinding | The investigators were not blinded to group allocations since all animal experiments were done after genotyping the mice. Therefore, blinding was not relevant to data collection. Nevertheless, the investigators were blinded during data analysis. |

# Reporting for specific materials, systems and methods

We require information from authors about some types of materials, experimental systems and methods used in many studies. Here, indicate whether each material, system or method listed is relevant to your study. If you are not sure if a list item applies to your research, read the appropriate section before selecting a response.

## Materials & experimental systems

| n/a | Involved in the study | |
|---|---|---|
| ☐ | ☒ | Antibodies |
| ☐ | ☒ | Eukaryotic cell lines |
| ☒ | ☐ | Palaeontology and archaeology |
| ☐ | ☒ | Animals and other organisms |
| ☒ | ☐ | Human research participants |
| ☒ | ☐ | Clinical data |
| ☒ | ☐ | Dual use research of concern |

## Methods

| n/a | Involved in the study | |
|---|---|---|
| ☒ | ☐ | ChIP-seq |
| ☒ | ☐ | Flow cytometry |
| ☒ | ☐ | MRI-based neuroimaging |

## Antibodies

| | |
|---|---|
| Antibodies used | Dilutions and source of the antibodies used in this manuscript are detailed in the method section.<br>GLUT2- Alomone labs, Cat#AGT022, lot#AGT022AN0102 |

GLUT2- Thermo Fisher Scientific, Cat#720238, lot#2133399
Phosphor-S6 (Ser240/244) (D68F8)- Cell Signaling Technology, Cat#5364
Phospho-Akt (Ser473)- Cell Signaling Technology, Cat#4058
SREBP1- Abcam, Cat#ab3259, lot#GR3277380-7
SREBP1- Abcam, Cat#ab193318, lot#GR3257438-7
SGLT2- Abcam, Cat#ab85626, lot#GR264147-3
CB1R- Immunogen, Cat#CB1, lot#BM01
Lrp2 / Megalin- Abcam, Cat#ab76969, lot#GR90326-1
β actin- Abcam, Cat#ab49900, lot#GR3283184-6
VCP- Abcam, Cat#ab204290, lot#GR3282909-2
SLC6A19- Abcam, Cat#ab180516, lot#GR147629-15
SLC7A5-Cell Signaling, Cat#5347S, lot#3
S6 ribosomal protein (5G10)- Cell Signaling Technology, Cat#2217
Akt- Cell Signaling Technology, Cat#9272
Fibrillarin- Abcam, Cat#ab4566, lot#GR3305870-2
DAGLα- Abcam, Cat#ab81984, lot#GR40151-41
DAGLβ- Abcam, Cat#ab191159, lot#GR275880-17
Goat Anti Rabbit IgG Alexa Fluor® 488- Abcam, Cat#ab150077, lot#GR266344-1
APC Donkey Anti Rabbit IgG- Jackson, Cat#711-136-152, lot#122435
Donkey Anti Mouse IgG (HRP)- Abcam, Cat#ab98799, lot#GR3296460-1
Donkey Anti Rabbit IgG (HRP)- Abcam, Cat#ab97085, lot#GR3218200-7

Validation

We used antibodies that were validated for species and applications by their respective manufacturer (listed below). Titration of all antibodies to determine optimal dilutions were validated in our lab. In addition, appropriate positive and negative controls (e.g. knock-out, knock-down, appropriate subcellular localization) were included in the relevant experiments.

GLUT2: https://www.alomone.com/p/anti-glucose-transporter-2-glut2/AGT-022
GLUT2: https://www.thermofisher.com/antibody/product/GLUT2-Antibody-Polyclonal/720238
Phosphor-S6: https://www.cellsignal.com/products/primary-antibodies/phospho-s6-ribosomal-protein-ser240-244-d68f8-xp-rabbit-mab/5364
Phospho-Akt: https://www.cellsignal.com/products/primary-antibodies/phospho-akt-ser473-193h12-rabbit-mab/4058
SREBP1: https://www.abcam.com/srebp1-antibody-2a4-ab3259.html
SREBP1: https://www.abcam.com/srebp1-antibody-ab193318.html
SGLT2: https://www.abcam.com/sglt2-antibody-ab85626.html
CB1R: https://www.immunogenes.com/catalogue-models/technology#anti-cb1-rabbit. We have validated this antibody for Western blot and used CB1R-KO mouse for negative control.
Lrp2/ Megalin: https://www.abcam.com/lrp2--megalin-antibody-ab76969.html
β actin: https://www.abcam.com/hrp-beta-actin-antibody-ac-15-ab49900.html
VCP: https://www.abcam.com/hrp-vcp-antibody-epr33072-ab204290.html
SLC6A19: https://www.abcam.com/slc6a19-antibody-epr14154b-ab180516.html
SLC7A5: https://www.cellsignal.com/products/primary-antibodies/lat1-antibody/5347
S6 ribosomal protein: https://www.cellsignal.com/products/primary-antibodies/s6-ribosomal-protein-5g10-rabbit-mab/2217
Akt: https://www.cellsignal.com/products/primary-antibodies/akt-antibody/9272
Fibrillarin: https://www.abcam.com/fibrillarin-antibody-38f3-nucleolar-marker-ab4566.html
DAGLα: https://www.abcam.com/dagla-antibody-ab81984.html
DAGLβ: https://www.abcam.com/daglb-antibody-c-terminal-ab191159.html

## Eukaryotic cell lines

Policy information about cell lines

| | |
|---|---|
| Cell line source(s) | human RPTEC-Renal Proximal Tubule Cells- Lonza, Cat#CC-2553<br>HEK 293 cells- ATCC, Cat#CRL-1573 |
| Authentication | Both cell lines were authenticated using morphology and PCR methods |
| Mycoplasma contamination | All cell lines were tested negatively for micoplasma |
| Commonly misidentified lines (See ICLAC register) | No commonly misidentified cell lines were used |

## Animals and other organisms

Policy information about studies involving animals; ARRIVE guidelines recommended for reporting animal research

Laboratory animals

Detailed description related to the animal housing is provided within the manuscript. Only male mice were used up to 16 weeks of age. The following strains were used in this study:
CB1Rfl/fl mice, Udi et al., 2017
RPTORf/f mice, Jackson Laboratory #013188
TSCfl/fl; Tsc2tm2.1Djk/Mmjax, Jackson Laboratories # 37154

| | |
|---|---|
| | GLUT2f/f mice, Seyer et al., 2013<br>iL1-sglt2-Cre mice, Rubera et al., 2004<br>AkitaIns2+/C96Y mice, Jackson Laboratory #003548 |
| Wild animals | The study did not involve wild animals |
| Field-collected samples | The study did not involve field-collected samples |
| Ethics oversight | The Institutional Animal Care and Use Committee of the Hebrew University (AAALAC accreditation #1285; Ethic approval number MD-19-15784) approved the experimental protocol used. Animal studies are reported, in compliance with the ARRIVE guidelines. |

Note that full information on the approval of the study protocol must also be provided in the manuscript.
