# Supplementary material for: Opposite physiological and pathological mTORC1-mediated roles of the CB1 receptor in regulating renal tubular function
Source: Nat Commun. 2022 Apr 4;13:1783. doi: 10.1038/s41467-022-29124-8 (PMC8980033; doi:10.1038/s41467-022-29124-8)
Supplement: Supplementary file 4 — Source Data [file 41467_2022_29124_MOESM4_ESM.zip › Source Data_uncropped gels_Figures.pdf]

Figure2a

Akita- RPTC$^{CB1R+/+}$ Akita- RPTC$^{CB1R-/-}$

pS6

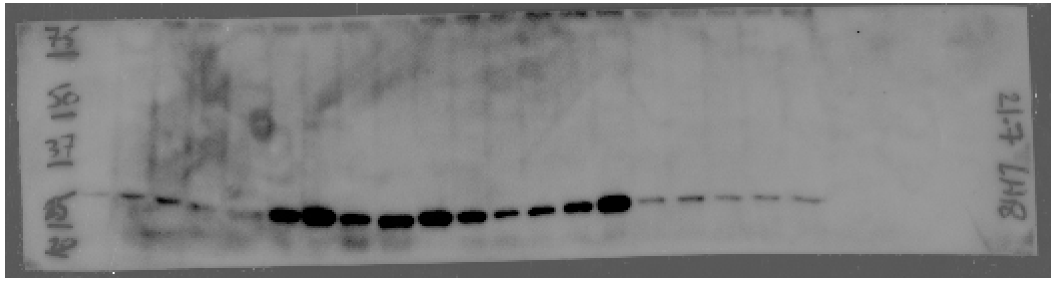

Akita- RPTC$^{CB1R+/+}$ Akita- RPTC$^{CB1R-/-}$

tS6

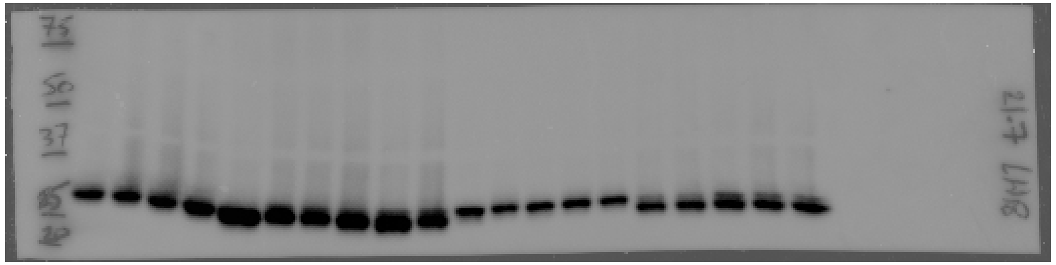

Figure2a, c

Akita- RPTC$^{CB1R+/+}$ Akita- RPTC$^{CB1R-/-}$

GLUT2

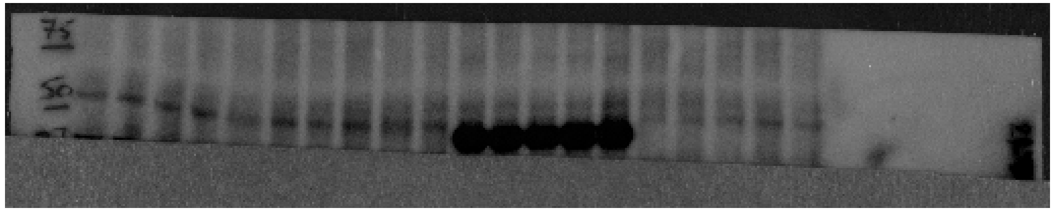

Akita- RPTC$^{CB1R+/+}$ Akita- RPTC$^{CB1R-/-}$

Actin

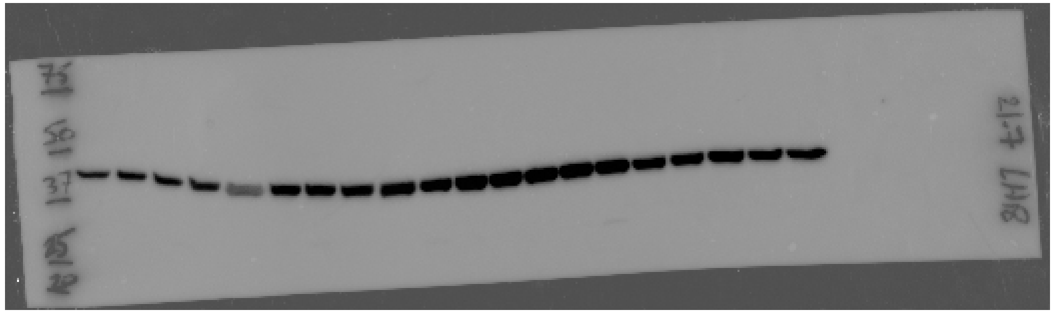

Figure2b

Akita- RPTC$^{CB1R+/+}$ Akita- RPTC$^{CB1R-/-}$

pAKT

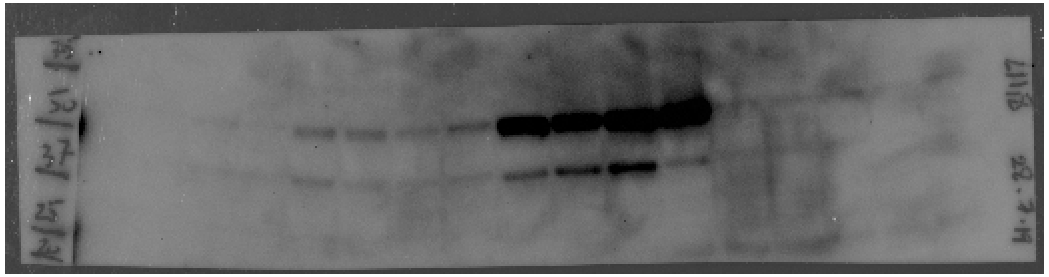

Akita- RPTC$^{CB1R+/+}$ Akita- RPTC$^{CB1R-/-}$

tAKT

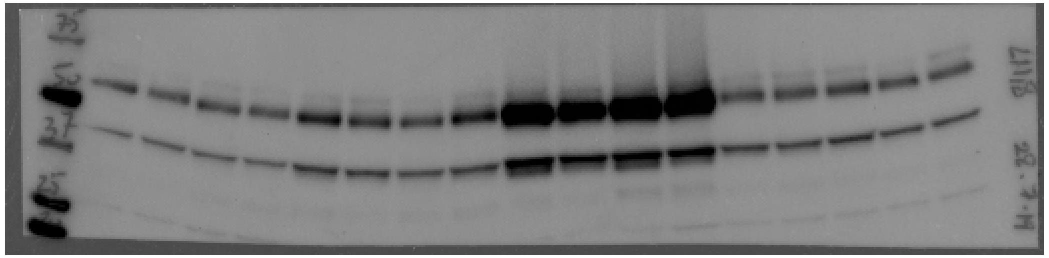

Akita- RPTC$^{CB1R+/+}$ Akita- RPTC$^{CB1R-/-}$

Actin

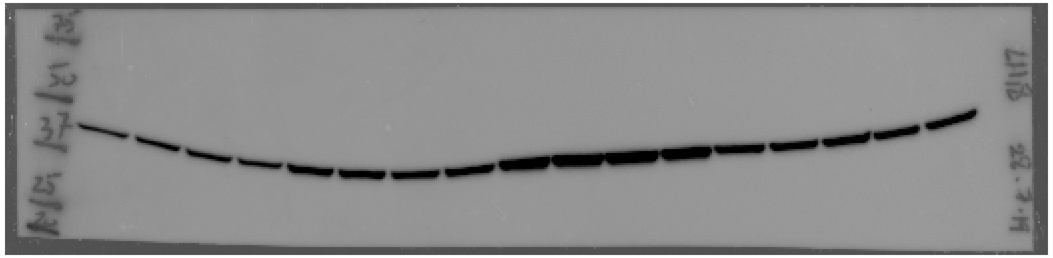

Figure 2j

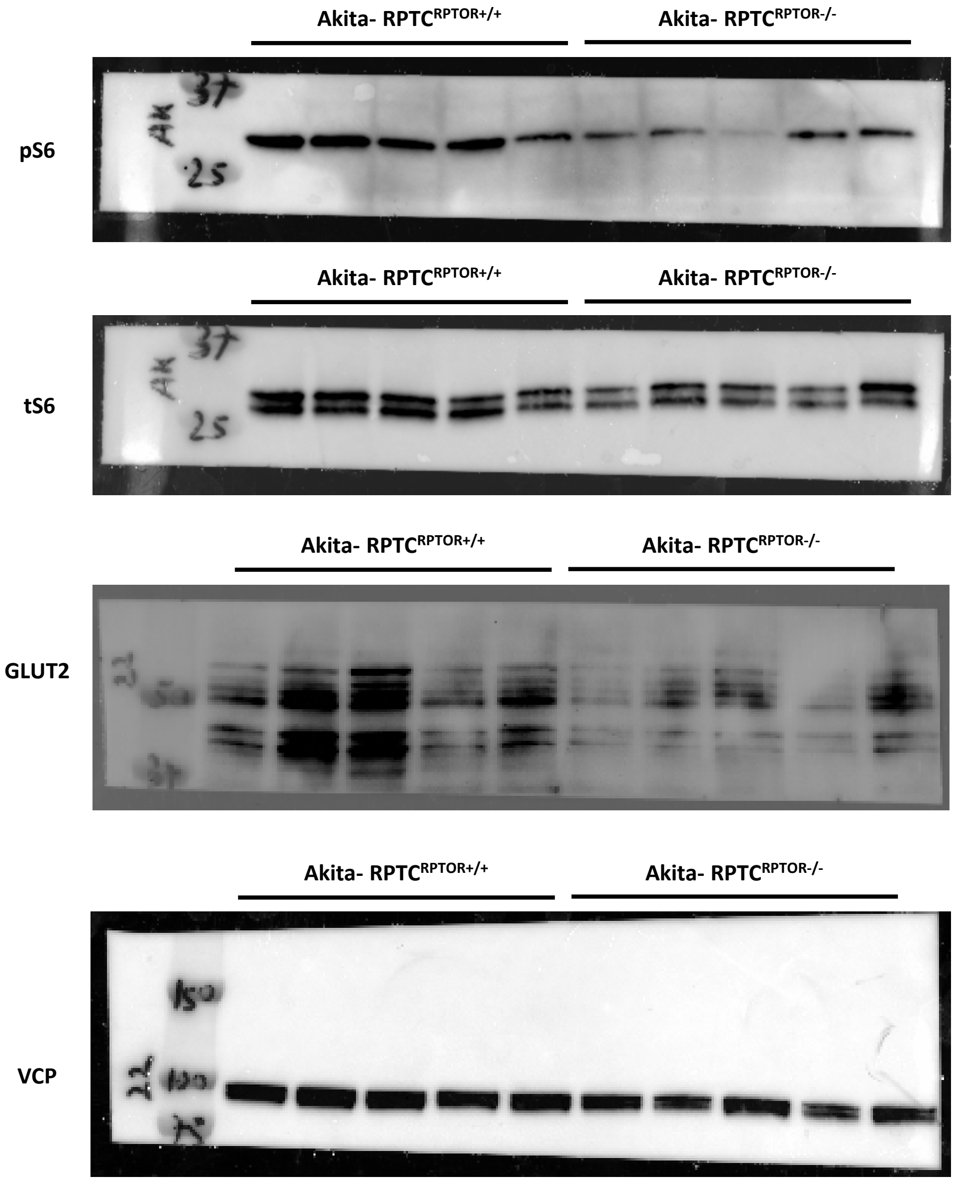

Figure 2n

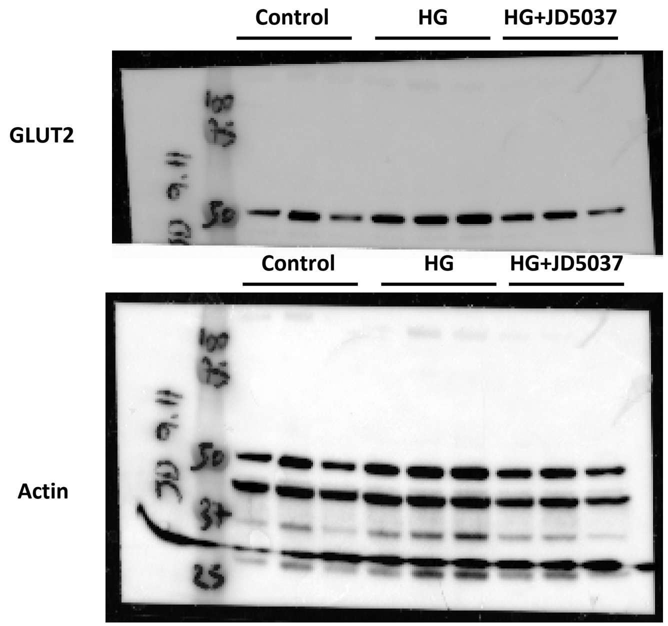

Figure 2u

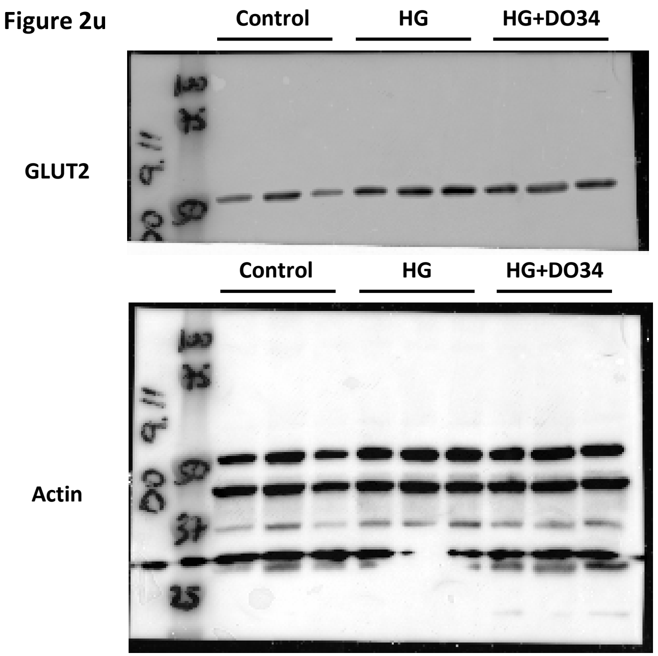

Figure 3a

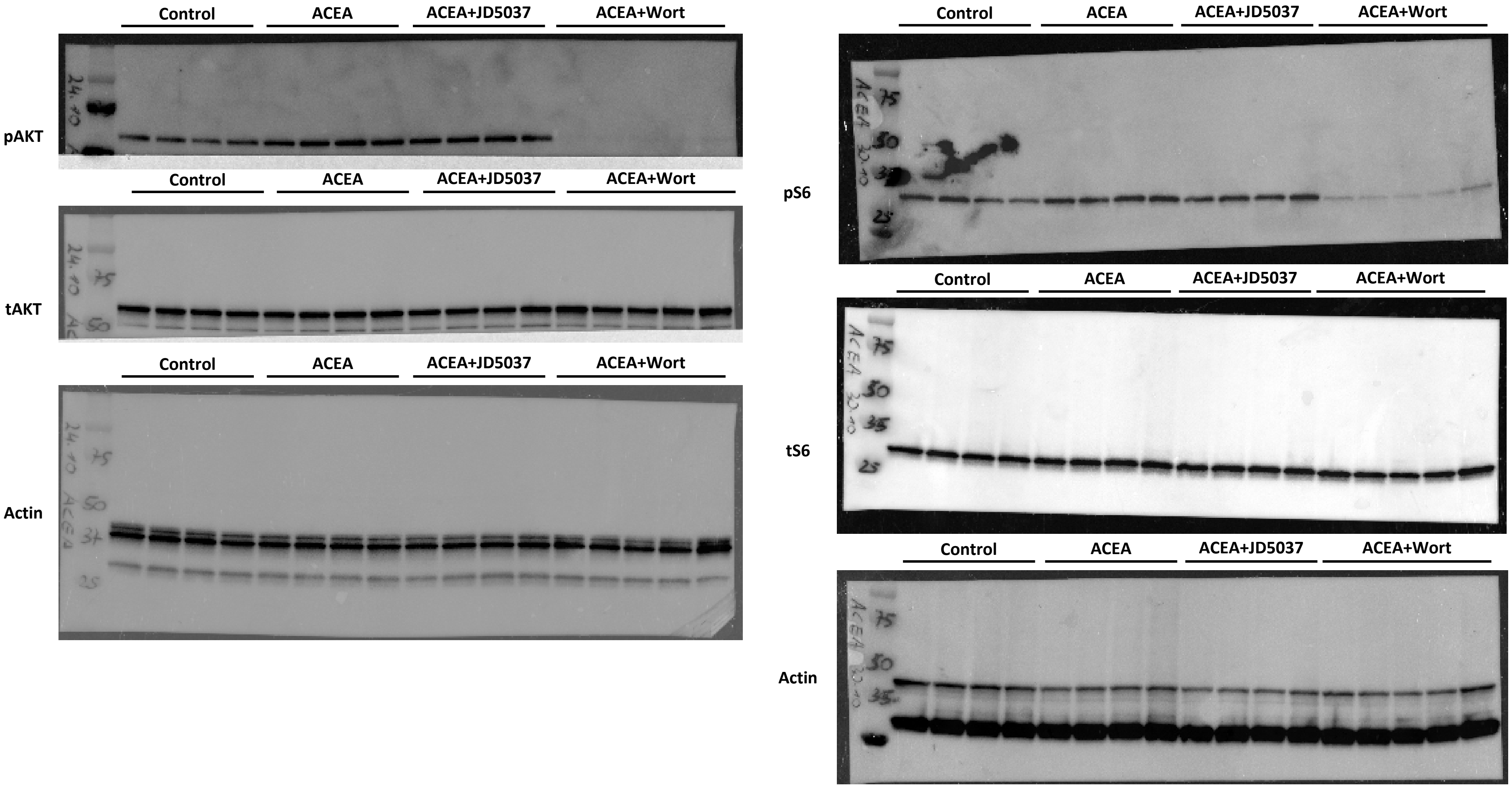
Control
ACEA
ACEA+JD5037
ACEA+Wort
pAKT
tAKT
Actin
pS6
tS6
Actin

Figure 3d

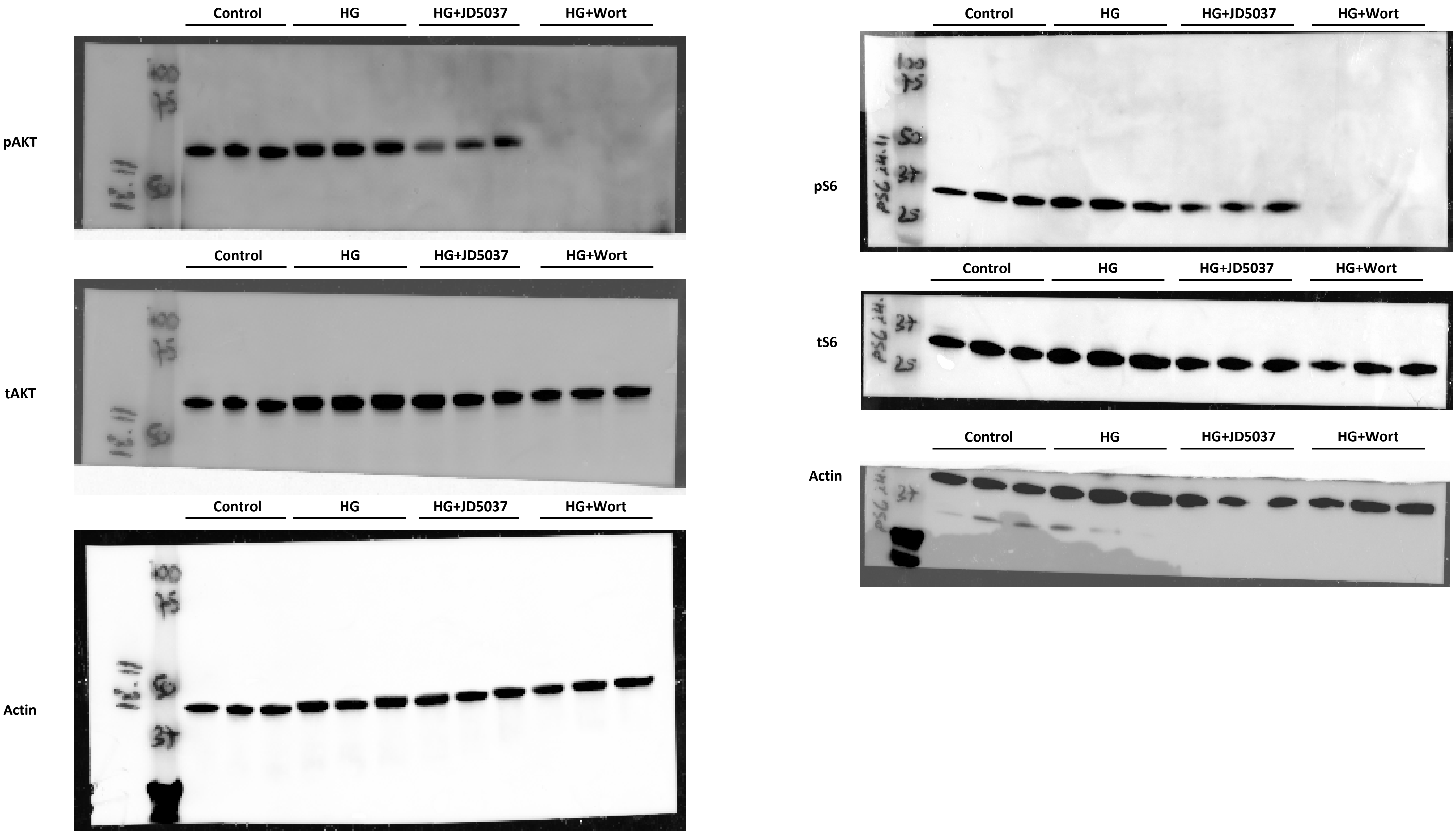

Figure 3g

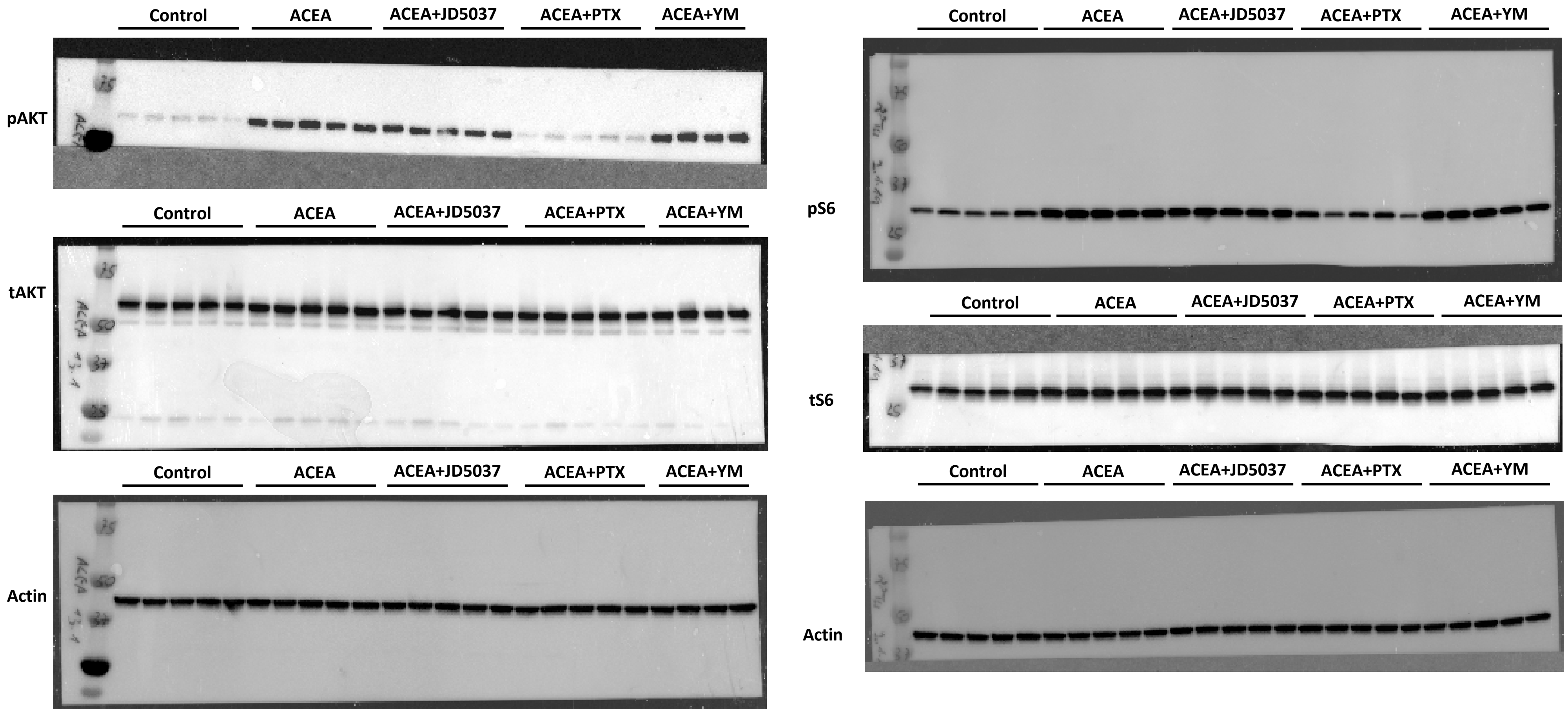
Control
ACEA
ACEA+JD5037
ACEA+PTX
ACEA+YM
pAKT
Control
ACEA
ACEA+JD5037
ACEA+PTX
ACEA+YM
tAKT
Control
ACEA
ACEA+JD5037
ACEA+PTX
ACEA+YM
Actin
Control
ACEA
ACEA+JD5037
ACEA+PTX
ACEA+YM
pS6
Control
ACEA
ACEA+JD5037
ACEA+PTX
ACEA+YM
tS6
Control
ACEA
ACEA+JD5037
ACEA+PTX
ACEA+YM
Actin

Figure 3j

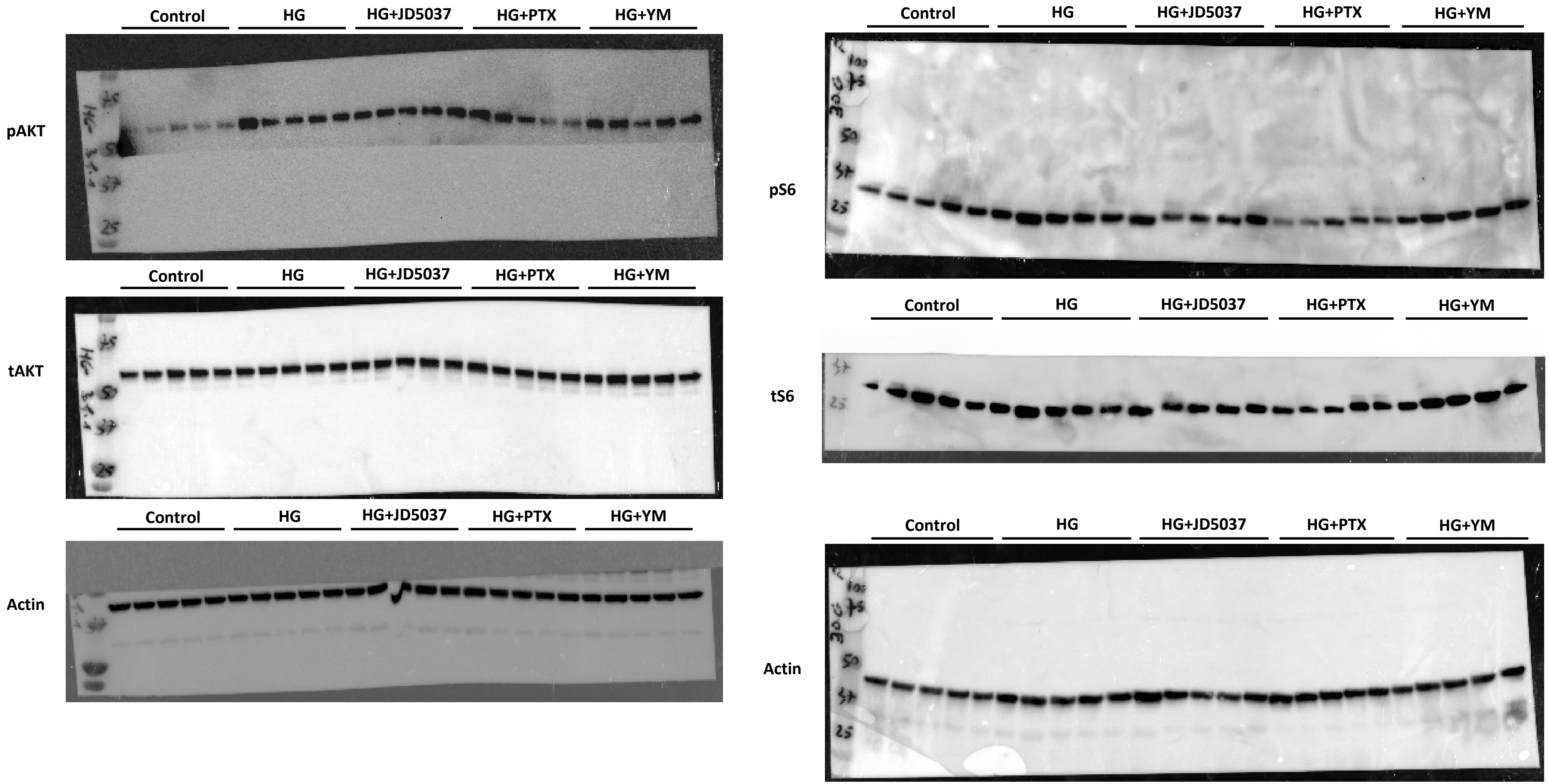
Control
HG
HG+JD5037
HG+PTX
HG+YM
pAKT
tAKT
Actin
pS6
tS6
Actin

Figure 4i

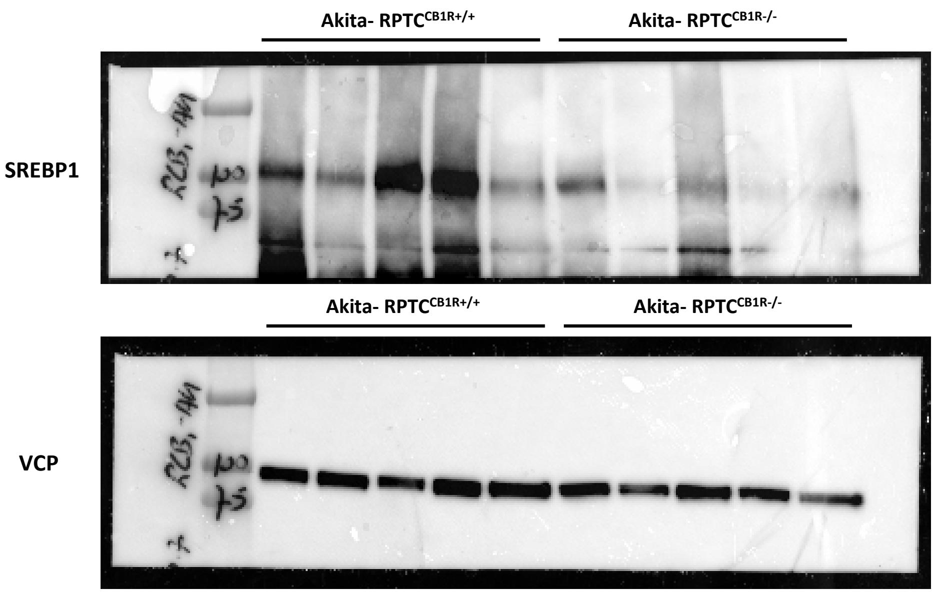

Figure 4l

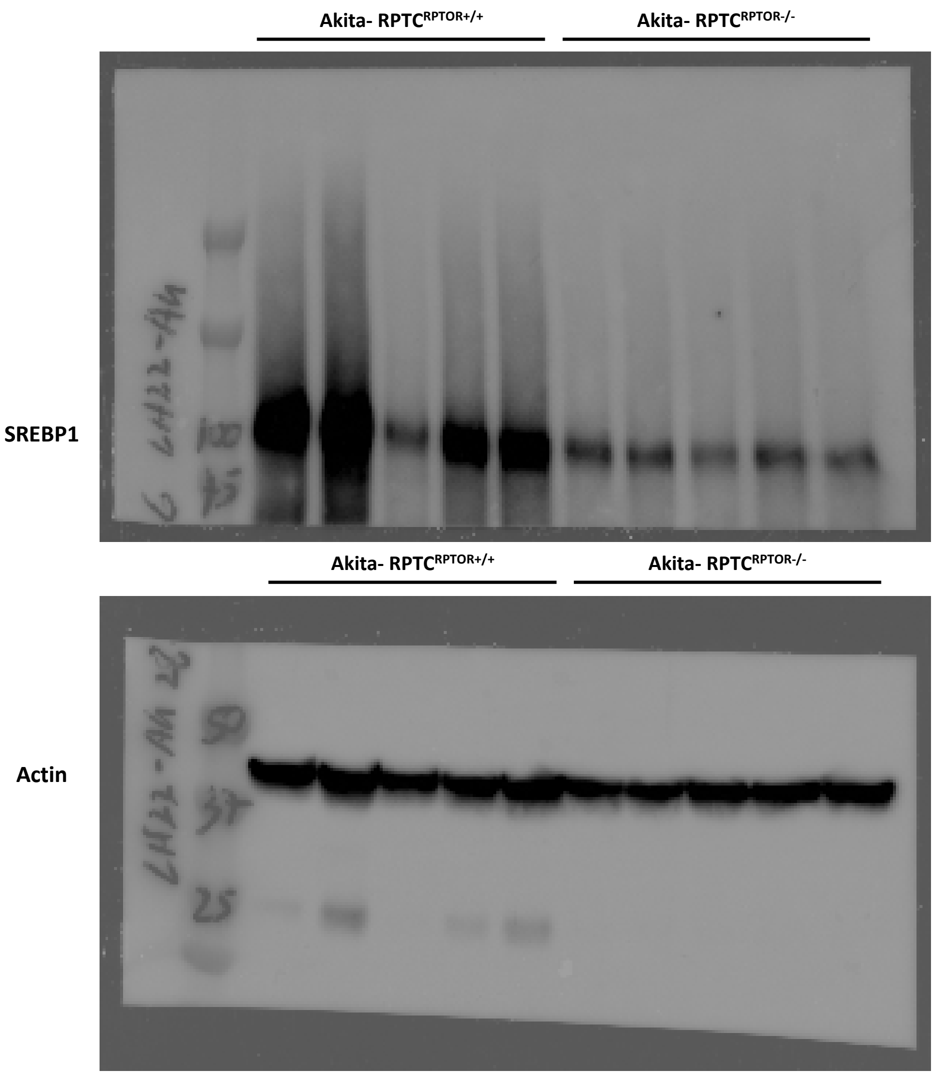

Figure 4n

Control | HG | HG+SREBP1 siRNA

SREBP1

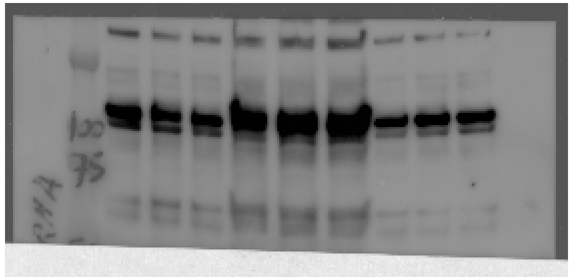

Control | HG | HG+SREBP1 siRNA

GLUT2

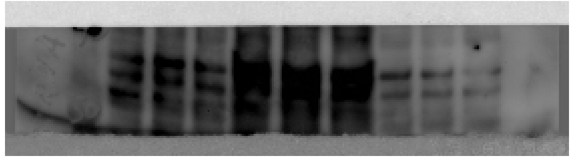

Control | HG | HG+SREBP1 siRNA

Actin

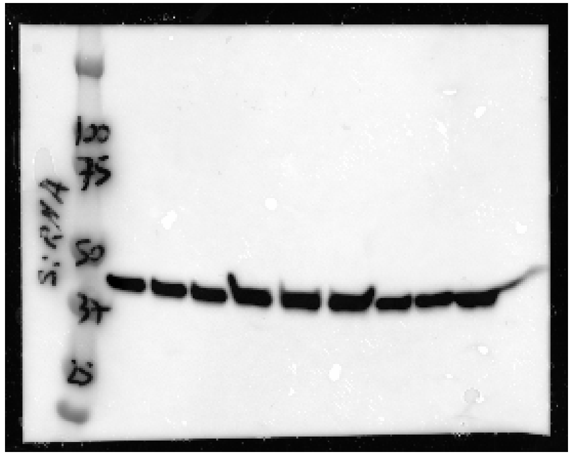

Figure 4s

Con | HG | HG+ JD5037 | HG+ RAP

SREBP1

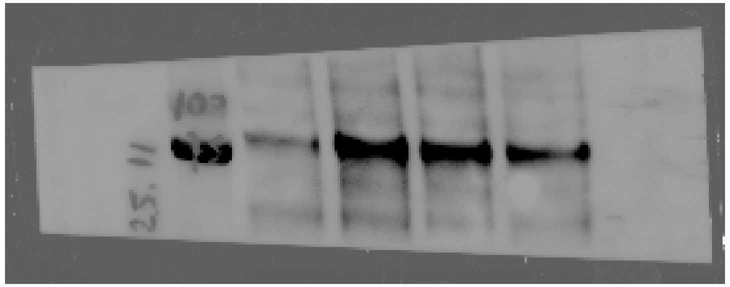

Con | HG | HG+ JD5037 | HG+ RAP

Fibrillarin

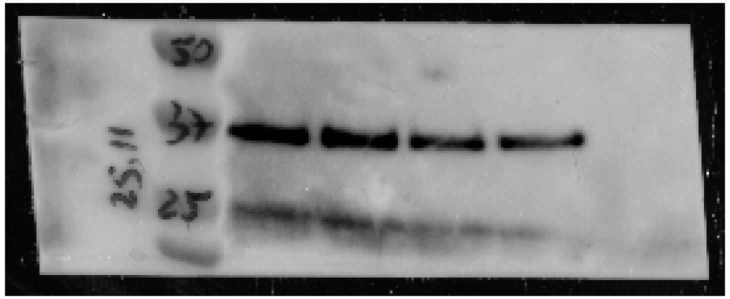

Figure 5a

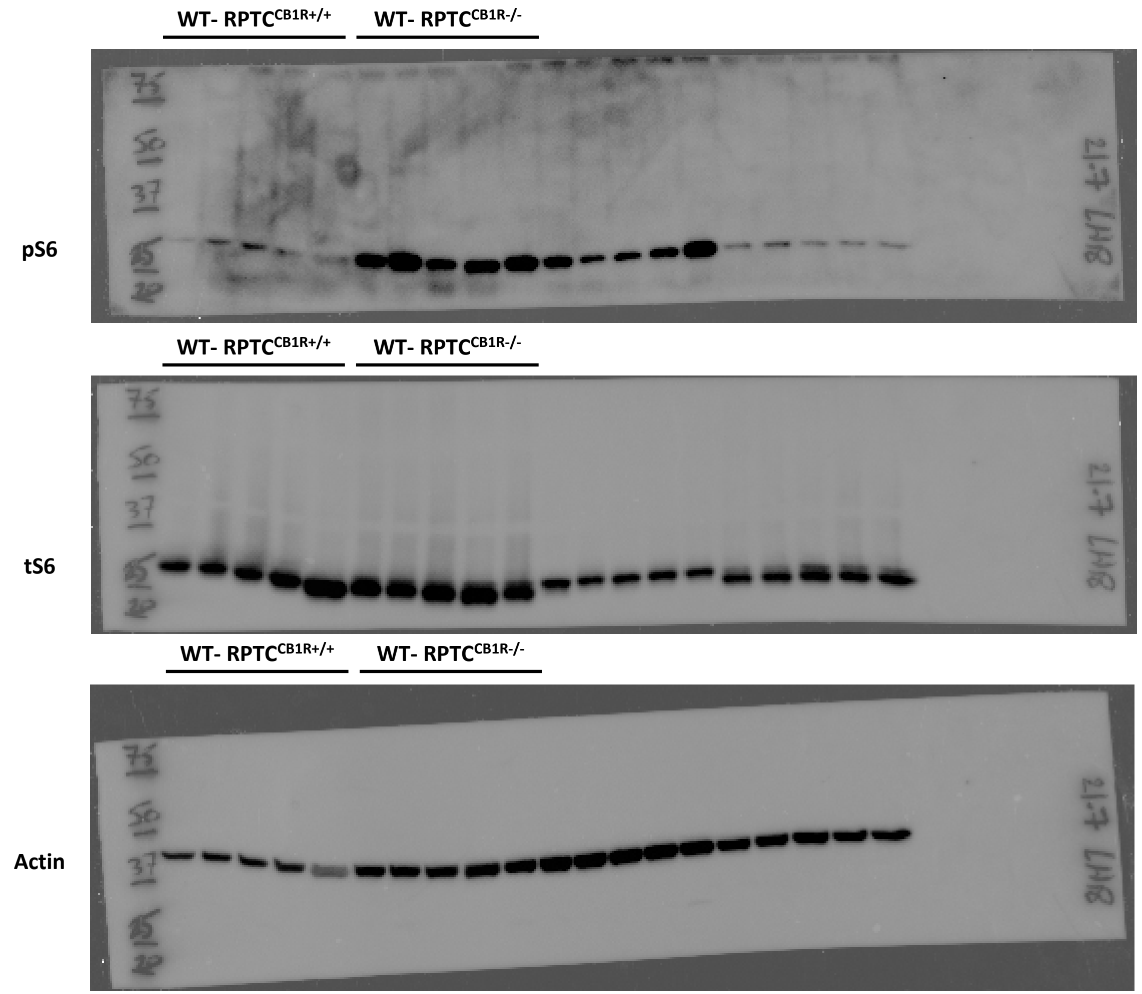

Figure 5b

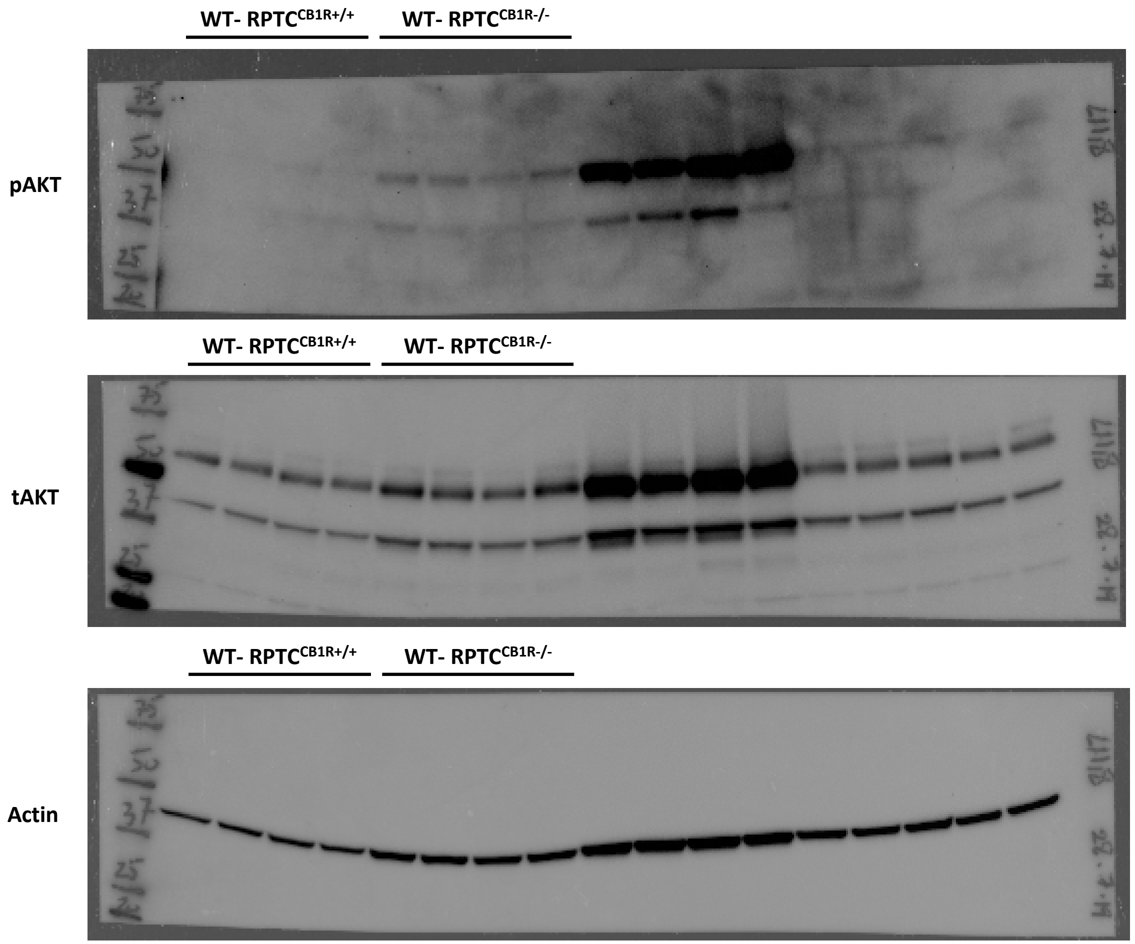

Figure 5e

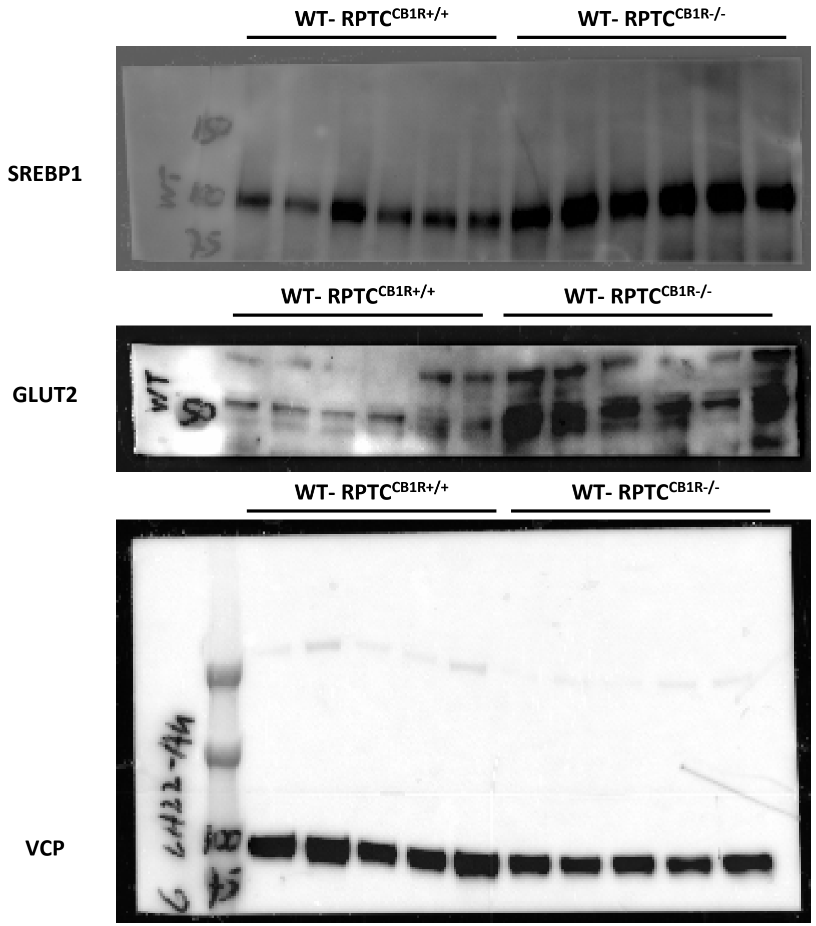
WT- RPTC$^{CB1R+/+}$
WT- RPTC$^{CB1R-/-}$
SREBP1
WT- RPTC$^{CB1R+/+}$
WT- RPTC$^{CB1R-/-}$
GLUT2
WT- RPTC$^{CB1R+/+}$
WT- RPTC$^{CB1R-/-}$
VCP

Figure 5h

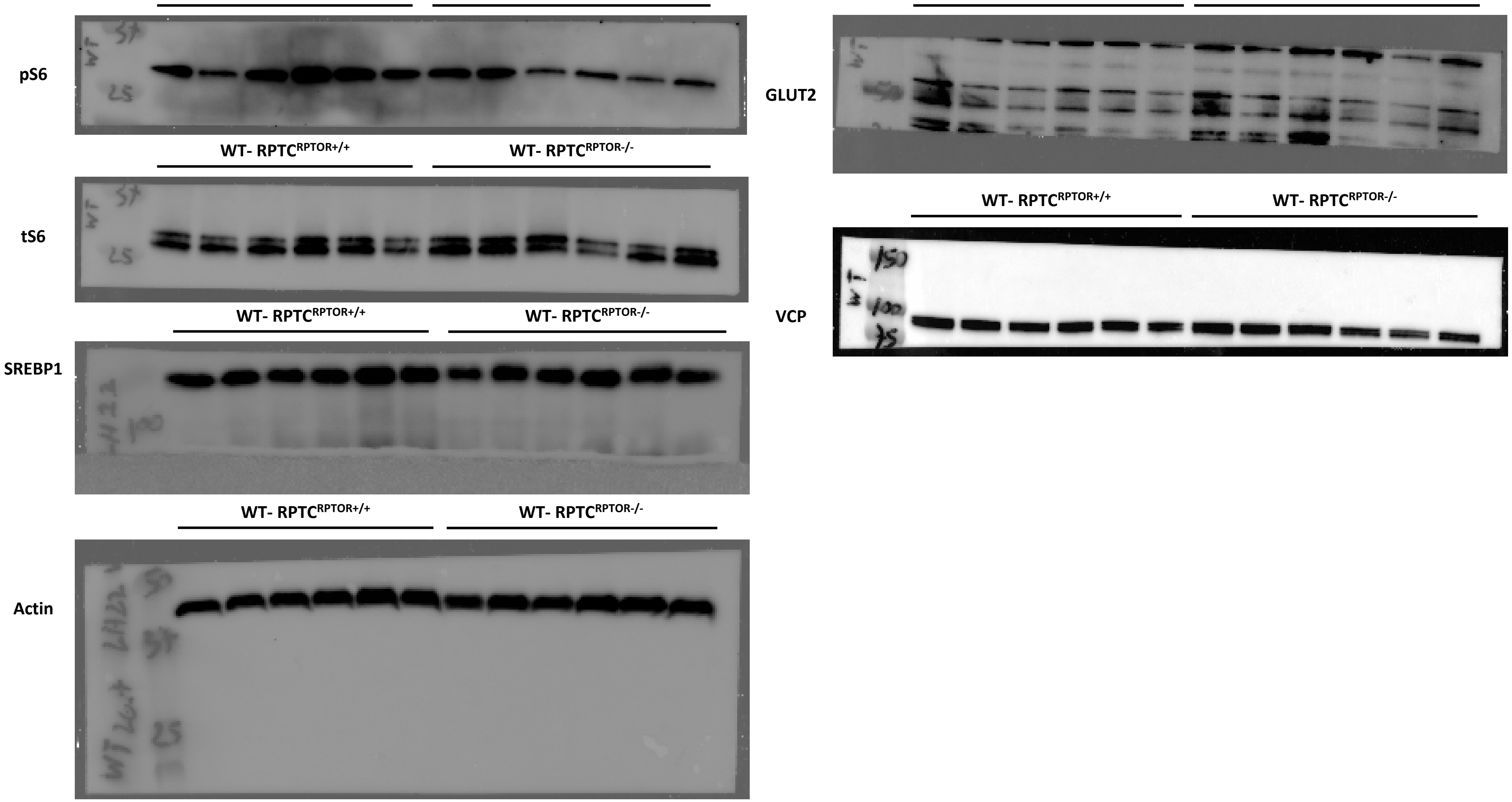

Figure 5m

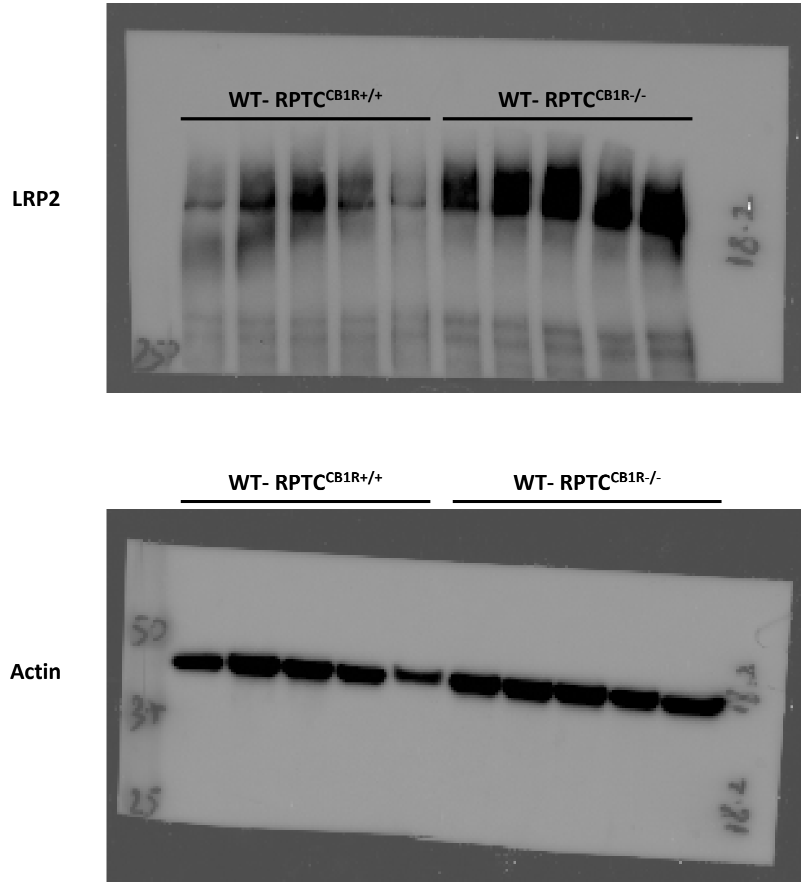

Figure 5r

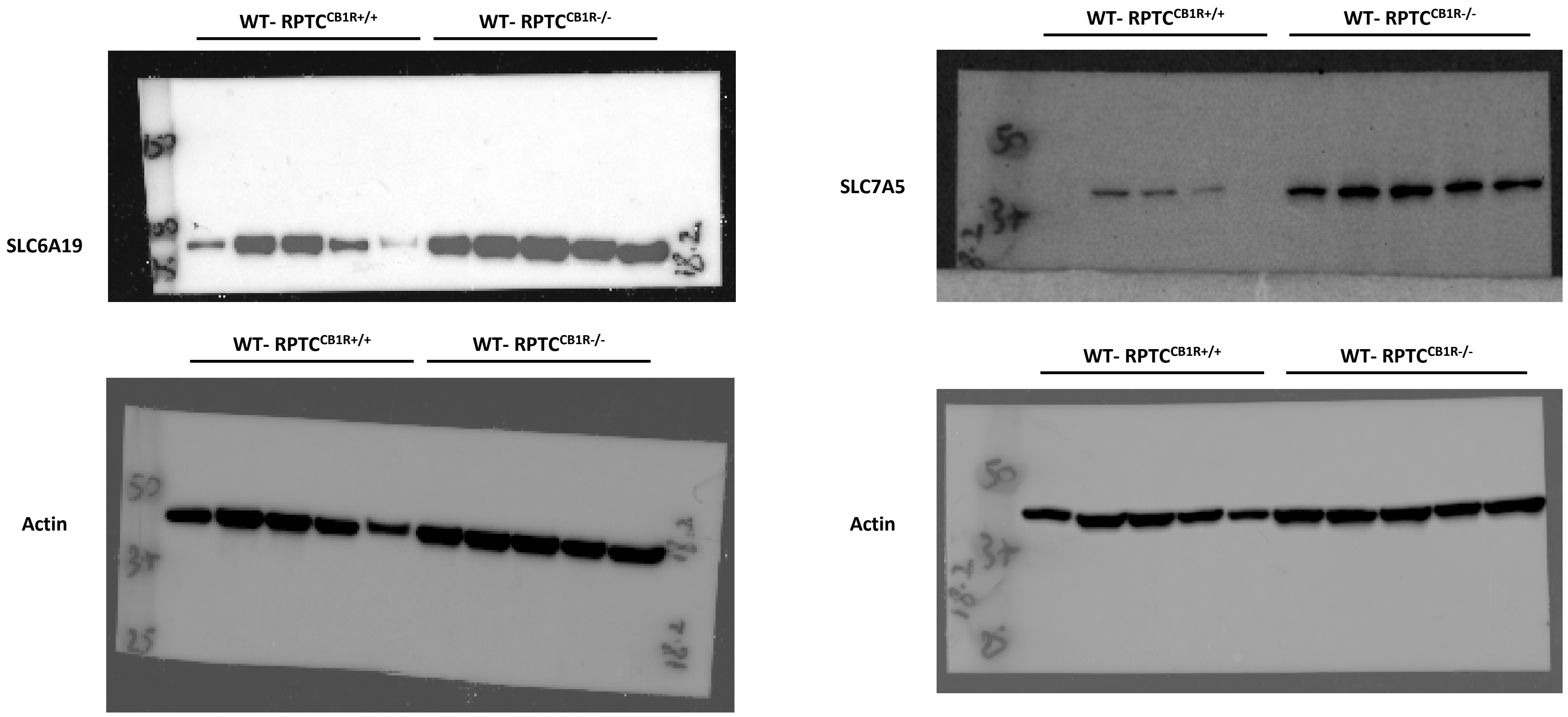
WT- RPTC$^{CB1R+/+}$
WT- RPTC$^{CB1R-/-}$
SLC6A19
WT- RPTC$^{CB1R+/+}$
WT- RPTC$^{CB1R-/-}$
SLC7A5
WT- RPTC$^{CB1R+/+}$
WT- RPTC$^{CB1R-/-}$
Actin
WT- RPTC$^{CB1R+/+}$
WT- RPTC$^{CB1R-/-}$
Actin
